# Characterizing paint technologies and recipes in Levantine and Schematic rock art: El Carche site as a case study (Jalance, Spain)

**DOI:** 10.1371/journal.pone.0271276

**Published:** 2022-08-15

**Authors:** Annalisa Chieli, Marius Vendrell, Clodoaldo Roldán, Pilar Giráldez, Ines Domingo

**Affiliations:** 1 LArcHer, Universitat de Barcelona, Barcelona, Spain; 2 Patrimoni 2.0, Barcelona, Spain; 3 ICMUV, Universitat de València, Valencia, Spain; 4 ICREA, Universitat de Barcelona, SERP, Barcelona, Spain; Universita degli Studi di Milano, ITALY

## Abstract

This paper contributes to current debates on the technologies and practices of prehistoric artists using the rock art site of el Carche (Jalance, Spain) as a case study. The site preserves both Levantine and Schematic paintings, yet poorly understood from an analytical point of view. In the past, it has even been argued how little differentiation there is between these two post-Paleolithic traditions in terms of paint composition. Our aim with this paper was to identify pigments, paint recipes and technologies and decipher the order of the superimpositions, both between Levantine motifs of different styles, and between these and the Schematic ones. To do so, we adopted a multi-stage and multi-technical analytical strategy, trying to find a balance between sound scientific investigation and impact on the art, considering the irreplaceable nature of this World Heritage rock art. As such, our approach begins with in situ non-invasive investigations using portable EDXRF, to then collect micro-samples for non-destructive analyses by means of Optical Microscopy, Scanning Electron Microscopy coupled with Energy Dispersive X-Ray Spectroscopy (SEM-EDX), micro-Raman Spectroscopy and Fourier Transform Infrared Spectroscopy (FTIR). One of the key highlights of these paper is the identification of up to four different paint compositions, produced with various hematite-based raw materials and different processing techniques. This variability had not been previously documented. Interestingly though, no direct correlations appear to exist between styles or sub-styles and recipes. Some of these paint mixtures were even shared by both traditions. These results are discussed in cultural terms, challenging previous interpretations suggesting a similar pigment composition between Levantine and Schematic art. Microstratigraphic analysis of the cross-sections only partially clarified the overlapping sequence unveiling the complexity of these analysis. They also revealed several degradation layers and external crusts related to rock alteration processes and biological formations. Their role in rock art conservation is also discussed.

## Introduction

In the last few decades, the application of various physico-chemical analyses to identify the composition of pigments of prehistoric rock art are enabling progress in our understanding of the technologies and practices of prehistoric artists worldwide and their evolution over time [1–4]. The identification of the mineral sources is used to explore mobility practices and social interactions [6–8]. Patterns of raw material transformation and variations in paint recipes are used to explore changing social identities and cultural practices and to identify authors, traditions and schools [5–8]. Stratigraphic analyses are important to record and characterize relationships of paint layers as well as natural coatings and order chronologically painting events [9,10]. This information would also help to understand processes of formation, change or alteration of the layers and to write the conservation history of prehistoric art.

Our current collective knowledge derives from the widespread use of these techniques to analyse various bodies of rock art around the world [1–4]. But progress is still needed to understand the specific cultural attributes of each tradition. Our aim with this paper is to contribute to both global and regional debates on this topic through the study of Levantine rock art [2,11,12]. Levantine rock art is a prehistoric artistic tradition specific to Mediterranean Iberia. Levantine panels draw attention to human figures, their activities, and their material culture through a series of naturalistic scenes. In these scenes, humans are depicted on their own or together with several animal species, to illustrate narratives of hunting, honey hunting, war and violence, maternity, and death, etc. (for details and debates on this art see for example reference [13]). This art dates sometime around 7500 years ago, even though no reliable numerical dates are known yet [14].

In the last few decades analytical approaches to study this tradition have mainly aimed at identifying the nature of the pigments and the recipes used to define the palette and the practices of Levantine artists [6,15–20]. Nevertheless, other issues, such as understanding the conservation threats and needs of both the paint and the substrate have also urge to use this type of analysis [6,9,10,15–25].

The number of Levantine sites analysed so far is still low (for a recent review see ref. [11]). Thus, more studies are needed to gain a better understanding of the technologies and practices of Levantine artists and their evolution over time and space, and to assess whether they share any common features with other major prehistoric artistic traditions.

To shed new light on these debates, this paper presents the results of our latest multi-stepped and multi-technical analytical approach to the study of rock art pigments of el Carche site. This site is of particular interest as it includes a variety of Levantine motifs in different painting shades and styles, coexisting also with Schematic motifs. Schematic art is an art depicting very simplified humans, animals, and geometric shapes, with different traditions across Europe. In Mediterranean Iberia this tradition dates between the Early Neolithic and the Bronze Age (for more details on this tradition in the study region see for example reference [26]). Even more interestingly, a good number of el Carche paintings are part of complex superimpositions, which are essential to construct a relative sequence of panel production. As such, El Carche provides the ideal setting to explore if the high level of stylistic variability observed in the motif shapes (whether Levantine or Schematic) is also seen in the composition of the paints used in their production. Our previous attempts to establish the sequence of motif addition at this site through non-invasive analysis (both *in situ* visual analysis and digital image analysis and processing techniques -DStretch and PCA-) were unsuccessful [27], as pigment opacity prevented the order of addition to be determined. Thus, the only option available to clarify the ordering of motif addition was sampling.

Following from this work, our next goal was to examine, identify and characterize the constituent materials of Levantine and Schematic paintings and explore correlations between them, both in terms of pigment composition and chronological sequences. In particular, our aims were:

- to characterize the nature of the pigments,
- to identify variations in raw materials and recipes among the different Levantine substyles, as well as between Levantine and Schematic art,
- to conduct stratigraphic analysis of the micro-samples to identify the sequence.

Considering the fragile and irreplaceable nature of prehistoric art, we decided to follow a multi-step and multi-analytical protocol. This protocol involved a preliminary scientific approach using non-invasive analytical method, followed by a more target sampling aimed at addressing our research questions while minimizing impacts on the art. During the initial phase of non-invasive in situ analysis we used EDXRF spectrometry for a first approach to the elemental composition of the pigments and the substrate and to guide subsequent sampling. This was followed by targeted micro-sampling of both single motifs and areas of overlap which were analyzed using a combination of different analytical techniques (Optical Microscopy, SEM-EDX, X-Ray Diffraction, Micro-Raman spectroscopy and FTIR). The characterization of the substrate was also included as a procedure to identify which components of the rock surface used as canvas were present in the pigment analysis and exclude them as potential components of the paint.

### Archaeological background and panel description

**Fig 1:**
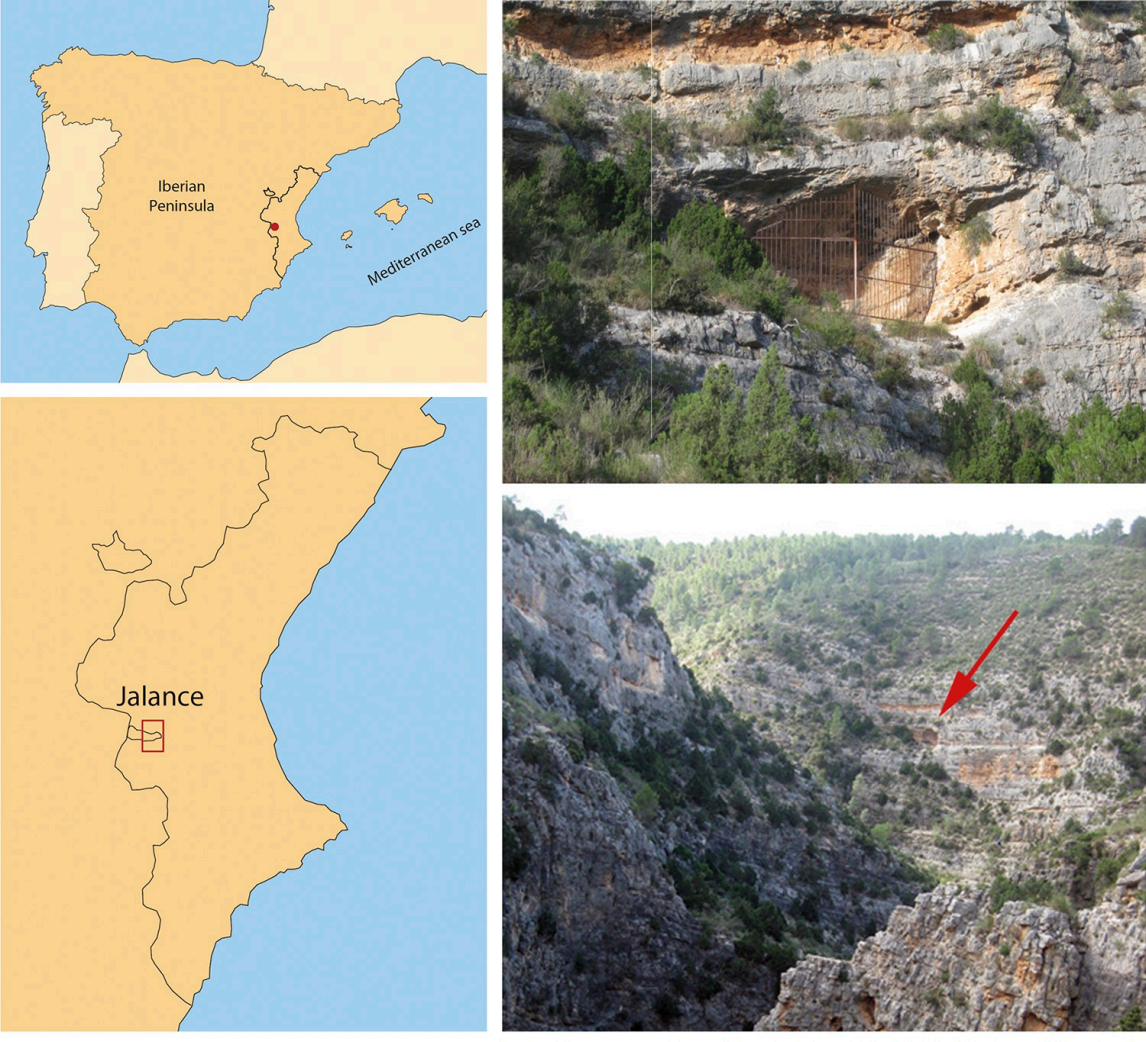
Geographical location of el Carche site (Jalance, Spain) and photographs of the site and the surroundings. Maps derived from ME500raster 2011–2012 CC-BY 4.0 ign.es.

El Carche rock shelter, also known as Fuente del Candil or Poveda rock shelter, after the name of the discoverer, is located in Jalance (Valencia province). It is a shallow rock shelter, measuring 7.29 m long and 4.43 m high and facing southwest in a quite strategic location, at the confluence between two ravines (for further details see ref. [28]) (Fig 1).

**Fig 2:**
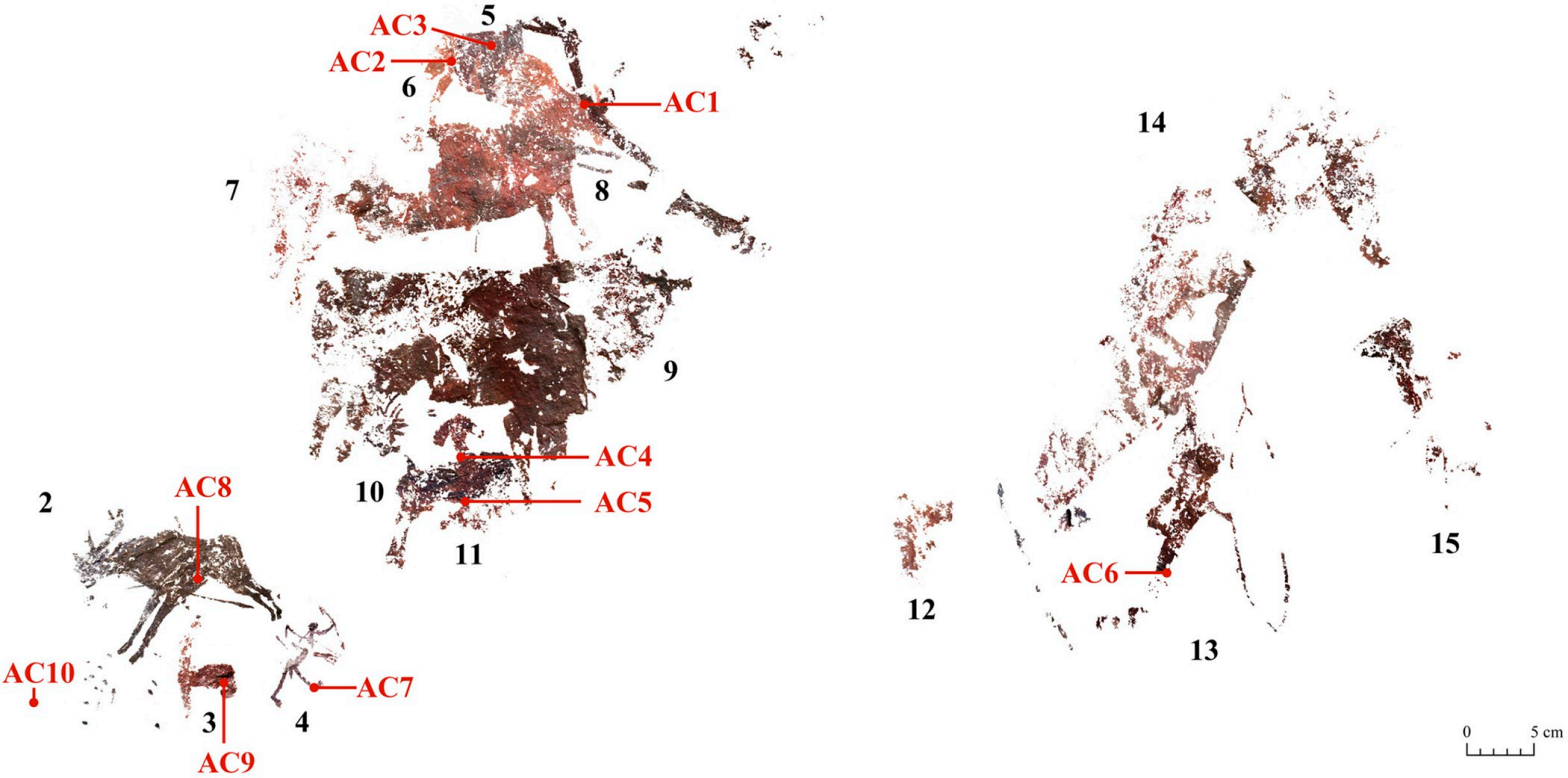
Digital tracing of the Levantine and Schematic paintings of el Carche rock shelter. Black numbers refer to motifs: 2. Deer, 3. Schematic unidentified quadruped, 4. Linear archer, 5. Human legs, 6. Unidentified quadruped, 7. Horse, 8. Potential remains of an animal, 9. Deer, 10. Deer, 11. Schematic anthropomorphic figure, 12. Archer, 13. Archer, 14 and 15. Unidentified quadruped. Red numbers refer to the points where microsamples were collected that are highlighted with a line. See Table 1 for further information on the microsamples.

The son of local historian Poveda discovered the site in 1997 [29]. Years later it was mentioned again by Aparicio [30]. But both publications were rather brief and superficial and did not reflect the special nature of the site for exploring questions related to the evolution of post-Palaeolithic rock art. In 2009, we started a series of research and knowledge transfer projects in collaboration with local city councils (Jalance and Ayora) and private archaeology consultants (Cavea Patrimonio Cultural). Our aim was to study the prehistoric art of the Ayora-Cofrentes Valley and to assess the potential to open some of these sites to the public. As part of these projects, we fully studied el Carche rock shelter, producing new graphic records of the art (photographs and digital tracings), and assessing the archaeological, artistic, and cultural values of this site [27,28]. As a result of these studies, we identified up to 15 painted motifs corresponding to two different post-Palaeolithic rock art traditions (Fig 2): Levantine rock art and Schematic rock art. Both traditions are listed in the Spanish National Heritage list as Bien de Interés Cultural (the maximum recognition and protection of a cultural asset in this country). In 1998, they were also included in the UNESCO World Heritage list, as a recognition of their exceptional universal values.

The inventory of Levantine motifs includes remains of several human and animal representations (for an in-depth description of the motifs and scenes see reference [28]):

- 4 humans of different styles and sizes likely involved in different hunting scenes: motif 4, a man of linear style with bow and arrows held in one hand and lifting a single arrow with the other, while walking to the right; 5, part of the legs of a human; 12, remains of a probable archer, possibly the head and an arrow; 13, archer facing right and holding a bow showing the bowstring. The legs are missing,
- 3 male deer of different styles, colors, and sizes, likely produced at different times: motif 2, pierced by an arrow through the belly and facing left. It was originally considered to be related to archer 4 as part of a hunting scene; 9, located in the center of the panel. It faces right, it is of large size, and it only preserves one of the antlers. 10, placed between the legs of deer number 9. It is significantly smaller, it faces left, and it is partially covered by a darker paint than may be related to a repainting event or to another almost lost figure,
- 1 horse facing right, with massive body and clear mane depicted in lighter red (motif 7),
- remains of other 4 unidentified quadrupeds (motifs 6 and 14 facing right and 15 facing left, and possibly 8, of which only a couple of parallel lines remain).

The list of Schematic motifs includes:

- a possible headless animal facing left (motif 3),
- a headless anthropomorphic motif with arched arms and legs (motif 11).

When we studied the site, we were first struck by the number of superimpositions among motifs. One of the overlaps includes up to seven of them: a superimposition including Levantine motifs 5, 6, 7, 8 and 9. The other one involves Levantine deer 10 and Schematic human 11 and, a third darker unidentified motif or repainting phase (as mentioned above), which has not been individually traced as it is difficult to isolate from deer 10’s body. This sort of palimpsests is quite unusual in Levantine panels. Superimpositions hold a particular scientific value for rock art researchers as they are key to contribute to current debates on the artistic sequence of Levantine art and the relationship between Levantine and Schematic traditions. While Schematic art seems to be well placed chronologically in the Neolithic and later periods, debates on the chronology and the lifestyles of Levantine artists (whether hunter-gatherers or farmers) are continuing (for a summary of these debates see for example references [31–34] among others). Therefore, clarifying the sequence between motifs 10 and 11 was important to this research, considering the potential chronological implications of the results.

## Materials and methods

### Sampling

Nine micro samples belonging to both Levantine and Schematic motifs located at el Carche rock shelter were collected. Sampling was performed by conservation expert E. Guillamet to minimize impact. Sampling was authorized by Generalitat Valenciana as required by the Spanish and Valencian heritage legislation (File number 2013/0609-V (SS.TT.). Thus, all necessary permits were obtained for the described study, which complied with all relevant regulations.

**Fig 3:**
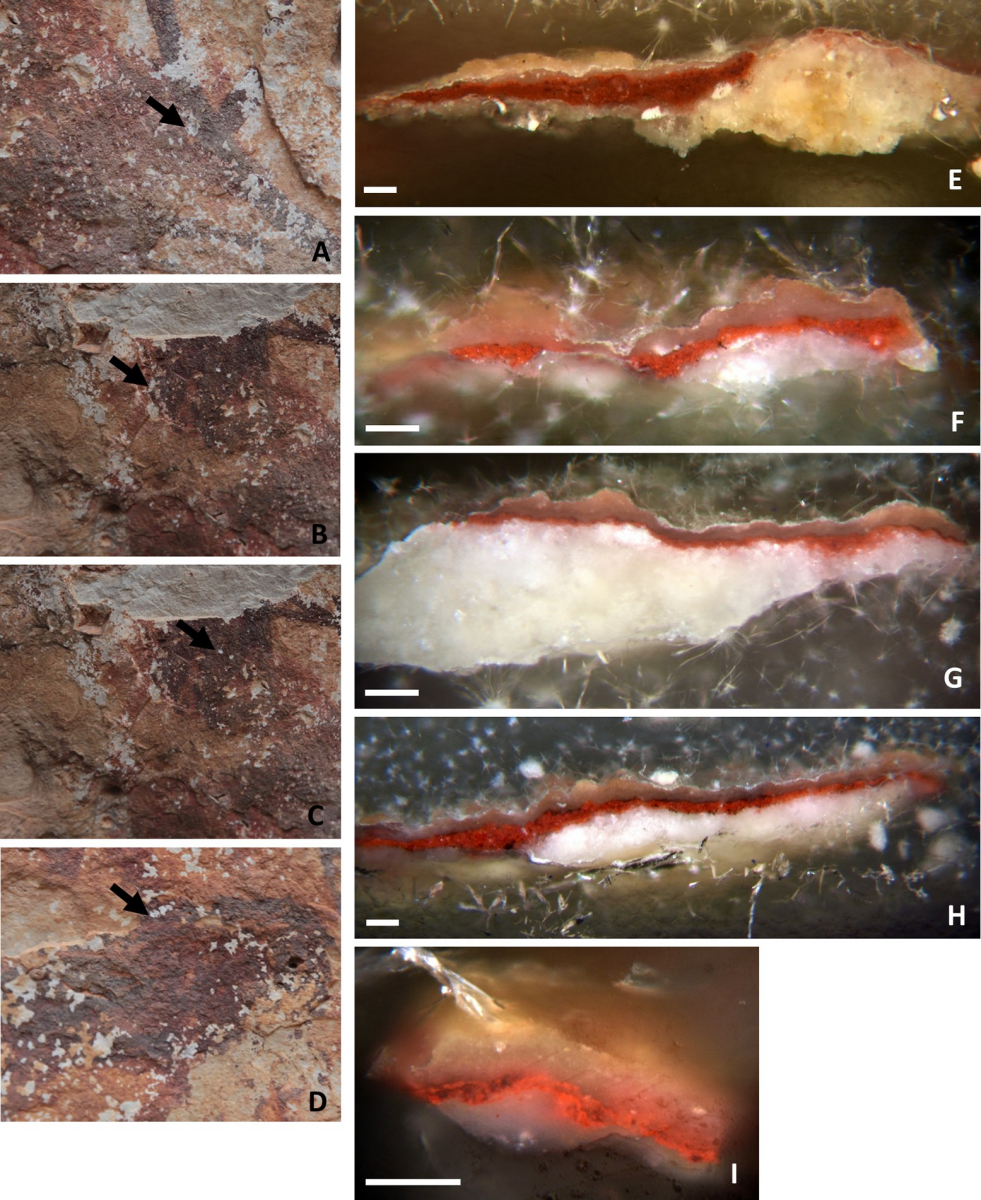
A-D: Location of the sampling areas before extraction. E-I: Optical microscope images of the cross-sections of the micro-samples collected in A-D. Images: A, E correspond to sample AC1; B, F and G correspond to samples AC2.1 and AC2.2; C, H correspond to sample AC3; D, I correspond to sample AC4. Scalebar = 100 μm.

**Fig 4:**
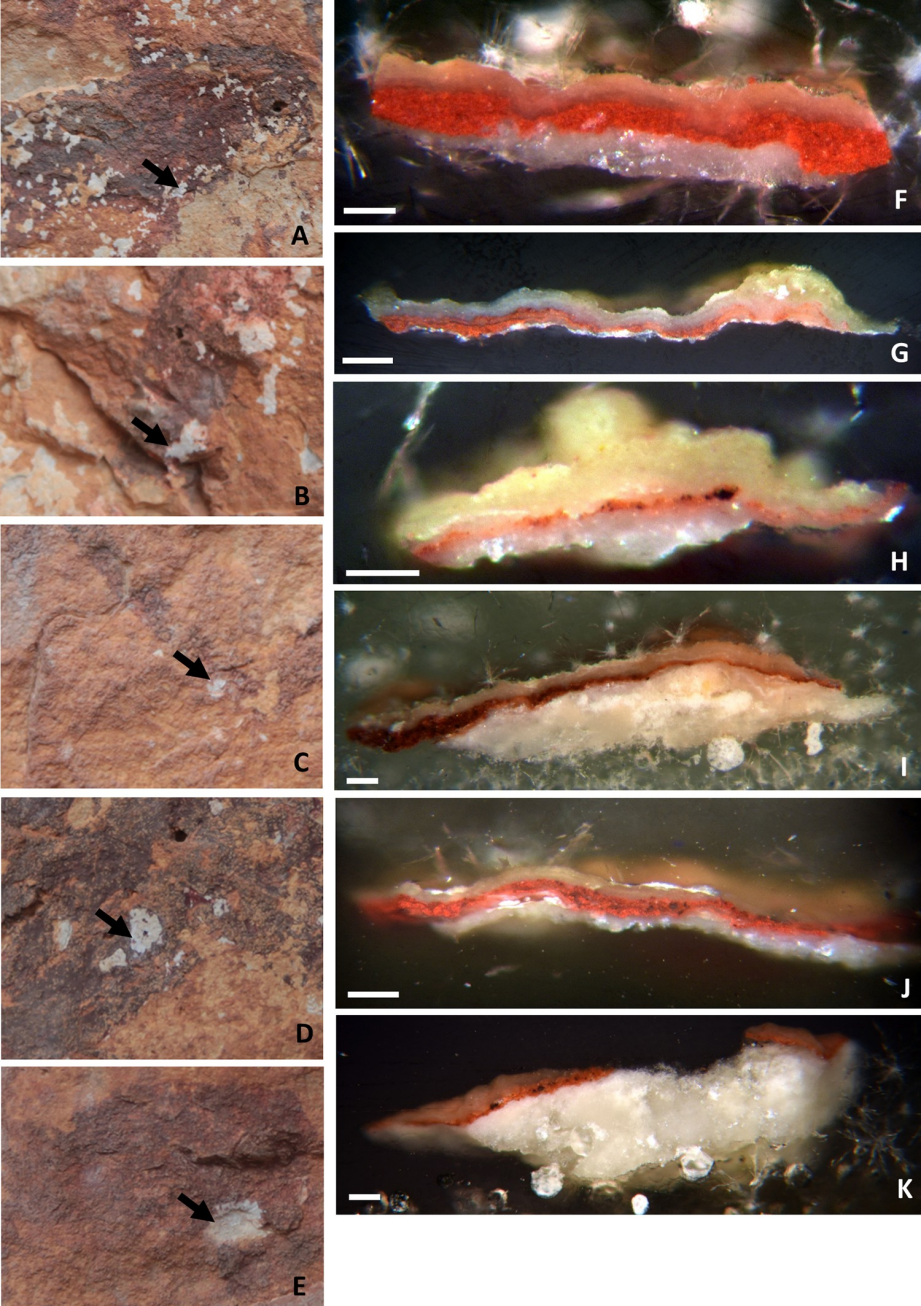
A-E: Location of the sampling areas before extraction. F-K: Optical microscope images of the cross-sections of the micro-samples collected in A-E. Images: A, F and G correspond to samples AC5.1 and AC5.2; B, H correspond to sample AC6; C, I correspond to sample AC7; D, J correspond to sample AC8; E, K correspond to sample AC9. Scalebar = 100 μm.

Samples include both single motifs (samples AC6 to AC9) and areas of superimposition (samples AC1 to AC5) to stablish the sequence of painting events (Table 1 and Figs 2–4). Only millimetric samples (size < 1 mm^2^) taken by professional conservators and prioritizing areas with existing lacunas (see Figs 3 and 4, left column) were taken. Our aim was to: i) minimize damages to the art and the rock surface and, at the same time, ii) avoid producing new fissures in the painted layer that eventually may contribute new sources of degradation. Microsamples were removed with sterile scalpel blades and stored in Eppendorf tubes. Sampled areas were photographically recorded before and after sampling. In the laboratory, the samples were embedded in TECHNOVIT 4001 acrylic resin to create cross-sections for in-depth examination of their stratigraphy and composition (see Figs 3 and 4, right column). The micro fragments appeared highly disaggregated due to a lack of cohesion between the constituent materials. Hence, some broke into smaller fractions, some of which were also embedded. Such is the case of samples AC2 and AC5, both resulting in two distinct polished cross-sections each (AC2.1/AC2.2 and AC5.1/AC5.2).

**Table 1:** List of micro samples collected from the motifs.

**Samples from superimposed motifs**	
**Sample ID**	**Motif Number, Description and Style**
**AC1**	**9,** dark purple deer, Levantine; **7,** red horse, Levantine; **6,** orange-like zoomorphic motif, Levantine
**AC2** *	**5,** fragment of dark purple human, Levantine; **6,** orange zoomorphic motif, Levantine
**AC3**	**5,** fragment of dark purple human, Levantine; **6,** orange zoomorphic motif, Levantine
**AC4**	**10,** dark purple deer, Levantine; **11,** purple/red anchoriform, Schematic
**AC5** *	**10,** dark purple deer, Levantine; **11,** purple/red anchoriform, Schematic
**Samples from single motif**	
**Sample ID**	**Motif Number, Description and Style**
**AC6**	**13,** red archer, Levantine
**AC7**	**4,** dark purple archer, Levantine
**AC8**	**2,** dark purple deer, Levantine
**AC9**	**3,** purple/red undefined motif, Schematic

*Samples with an asterisk are composed by two different fragments, namely AC2.1/AC2.2 for cross-section AC2 and AC5.1/AC5.2 for cross-section AC5.

Two samples from the substrate where also collected to differentiate between paint and rock surface materials. Sample AC10 was taken directly from the substrate next to motif 2, and it was analyzed without being embedded (S1 Fig 1 in S1 File, left column). Sample AC11 is a fragment detached from the wall (thus collected on the ground) and it preserves the external orange/brownish crust characteristic of the exposed rock surface (S1 Fig 1 in S1 File, right column). This sample was embedded in polyester resin.

### Analytical methods

#### Portable Energy Dispersive X-ray Fluorescence Spectroscopy (EDXRF)

**Fig 5:**
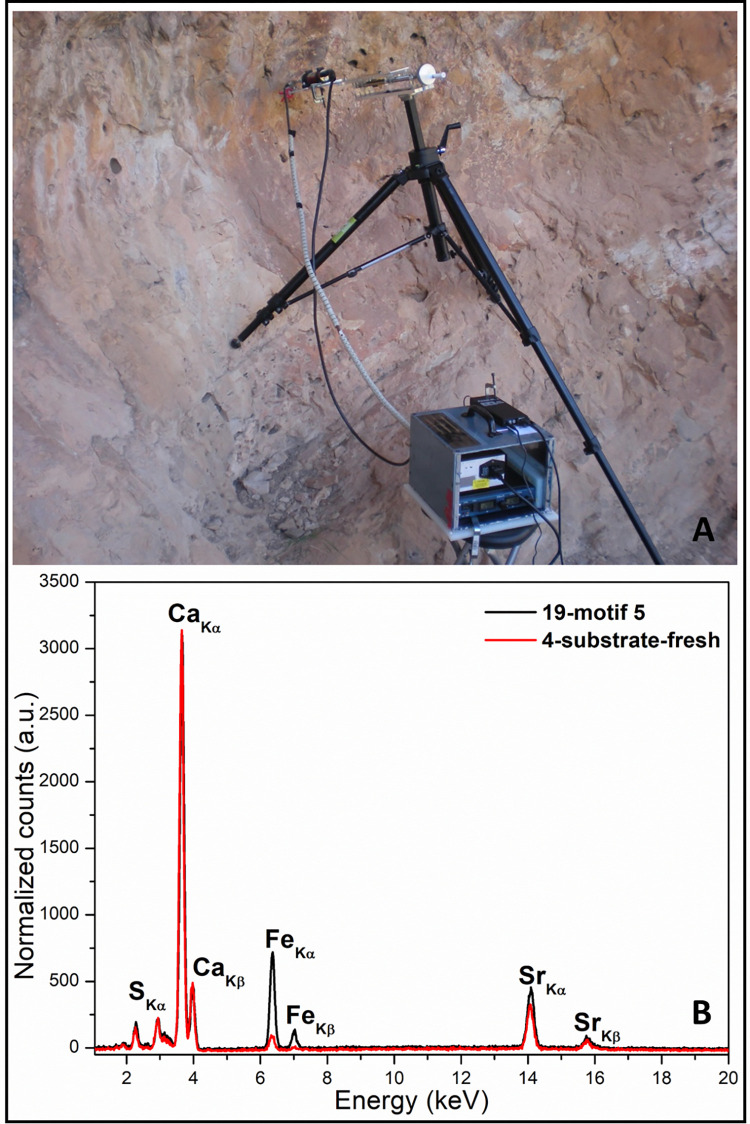
A) Portable EDXRF spectrometer employed for in-situ analyses; B) EDXRF spectra collected on the rock surface (red line) and on the red paint of motif 5 (black line).

The EDXRF spectra were recorded in situ using a portable spectrometer [19] composed by a) miniaturized X-ray tube (Eclipse-II Oxford Instruments) with transmission anode of silver (Ag) and that can operate up to 30 kV and 0.1 mA; b) a semiconductor detector Si-PIN (Amptek XR-100CR) of 6 mm2, 500 μm thick, with a 13 μm beryllium window and a resolution of 170 eV (FWHM @ 5.9 keV); c) a standard electronics chain with an MCA pocket 8000A multichannel analyzer (Amptek); d) a mechanical support designed to allow XYZ displacements and configure the angular geometry between tube, sample and detector (Fig 5A). A gasoline-powered 600 W alternating current electric generator was used as power source. The analyzes were carried out with the normal incidence of the X-ray beam on the sample and keeping the detector at an angle of 45° with respect to said normal and with an excitation-detection time of 200 s. The distance between sample and detector was about 2 cm. An aluminum collimator, located at the outlet of the tube, provides a 5 mm diameter irradiation area on the sample. The penetration depth assuming a density of 5 g/cm^3^ irradiated with a continuum X-ray spectra of 30 kV with a maximum of 20 keV, is about 300 microns for I/I_0_ = 10%. Thus, considering the thickness of the stratigraphy, the EDXRF spectra include information of all the strata (substrate included). The XRF spectra recorded were processed using the PyMCA software [35]. In particular, the fluorescence lines net areas of the most frequent element identified (values reported in S1 Table 1 in S1 File), have been divided by the total area of the spectrum to normalize the possible effects of the tube-sample-detector geometry and the fluctuations of the X-ray tube current intensity. Assuming the homogeneity of the substrate, the analyses were performed by recording spectra of both painted and unpainted areas to identify the elements of the pictorial layer and those of the substrate. 12 motifs were selected for EDXRF analysis, resulting in 33 non-destructive punctual analyses, of which 24 correspond to red pigments and 9 to the rock surface (S1 Table 1 and S1 Fig 1 in S1 File).

#### Optical microscopy

A Zeiss universal optical microscope with reflected light arrangement was used. A prism instead of a mirror was used as reflector to increase the light efficiency. The illumination was under dark field by observing the sample between crossed polarizers to cut the light reflected on the surface and to get only the diffuse reflectance inside the sample: this configuration allows to see the true colors of the layers forming the sample and thus, to study the stratigraphy layer by layer. Under these conditions, microphotographs of the samples were taken at different magnifications (8x, 16x and 40x).

#### Scanning Electron Microscopy–Energy Dispersive X-Ray Spectroscopy (SEM-EDX)

We used a Hitachi TM-3030plus with an acceleration voltage of 15 kV equipped with detectors of secondary and backscattered electrons as well as the X-ray dispersed by the sample (EDX) (Bruker, Scan Generator). Since this microscope works at low vacuum, the samples do not need to be metalized (and subsequently cleaned to remove the coating) and they can be observed and analyzed as they appear. This way we remove any risks of altering the samples.

#### X-Ray Diffraction

XRD analyses were exclusively performed in substrate sample AC10. A Bruker D8-Advance diffraction system operating at 40 kV and 30 mA with monochromatic Cu–Kα radiation was used. The samples were powered in an agate mortar and mounted in conventional aluminum holders before measurements. The intensity was measured in the 2θ range from 5° to 80° at a step size of 0.02° and a counting time of 0.2 s per step. The data were recorded and identified using PANalytical X’Pert HighScore Plus software and the diffractogram was plotted using OrignLab.

#### Micro-Raman spectroscopy

Raman spectra were obtained on polished cross-sections with a HORIBA Jobin Yvon Lab Ram HR800 dispersive spectrometer equipped with an Olympus BXFM optical microscope at the Molecular Spectroscopy unit at the CCiTUB. Two different excitation lasers have been used to investigate the micro samples, namely 523 nm and 785 nm, using a grating of 600 and 300 and a spectral resolution of ca. 1,7 and 1,6 cm^-1^, respectively. A 50× and 100x objective lens were used to focus the laser beam spot on the sample surface, with a spot size of ca. 2 and 1 μm respectively, using a laser power < 1 mW—to avoid any effect due to laser power induced transformation on the studied materials [36]- 5–10 s of exposure time and a number of spectra accumulations between 2–5. The spectra were recorded in the spectral range between 190–1650 cm^-1^ and they were plotted using OriginLab software without further data treatments.

#### Fourier Transform Infrared Spectroscopy (FTIR)

FTIR measurements in reflectance mode were obtained using a Nicolet™ iN10 MX Infrared Imaging Microscope, equipped with an MCT (mercury-cadmium-telluride) detector. For the mapping analyses of cross-sections AC1-AC9, the spectra have been collected over the 4000–675 cm^−1^ range, and they were obtained by averaging 64 acquisitions with 4 cm^−1^ spectral resolution, using an optical aperture of 100 μm with 5 μm of step-size. Surface of sample AC10 was analyzed using punctual system, following the same experimental conditions. Software OMNIC Picta (Thermo Fisher Scientific) was used to process the spectra and the imaging, and OriginLab to plot the selected curves. For the cross-sections analyses, we faced various difficulties with the current method to obtain the maps, both related to the penetration of the resin into the samples and their rough surfaces. Sample AC4 and AC6 were not analyzed due to their small dimension. The reflectance spectra acquired during the cross-sections analyses were directly converted to absorbance using the inbuilt OMNIC Picta Kramers–Kronig Transformation (KKT) to facilitate their reading [37,38]. These analyses have been performed at the Molecular Spectroscopy unit of the CCiTUB.

Measurements of cross-section of sample AC11 were performed by punctual ATR system using LUMOS II spectrophotometer by Bruker (spectral range from 600–4000 cm^-1^, 4 cm^-1^ spectral resolution) interfaced with OPUS software.

## Results

The results of the analyses will be discussed in two sections. The first one describes those analyses performed to characterize the substrate, while the second reports the analyses of the rock paintings and their cross-sections. As previously mentioned, the analysis of the substrate is a crucial prerequisite for a correct understanding of the results of the pictorial layers, which is the aim of this study. In both cases, non-invasive in situ EDXRF analyses were applied, followed by specific non-destructive methods.

### Characterization of the substrate

#### In situ non-invasive EDXRF analyses

EDXRF spectra recorded on the rock surface (S1 Fig 2 in S1 File) presented a typical composition of limestone rock with a massive presence of Ca as main element, followed by Sr and S. These elements could be associated with calcium and strontium sulfates (Fig 5B–red line). Comparing the different spectra of the rock surface, S amount is variable and such variation may correlate with the major/minor presence of external sulfate-based formations, likely gypsum, either as accretion or alteration products of the limestone substratum [16,25]. Minor presence of Fe, K, Ti, Mn also occurred in all analyzed points (S1 Table 1). The calculated fluorescence line areas of the most significant elements (S, K, Ca, Ti, Mn, Fe, and Sr), representative of the composition of all the analyzed points, and their codes, are displayed in S1 Table 1 in S1 File.

#### XRD and FTIR analyses of substrate samples

Substrate sample AC10 was analyzed by X-ray diffraction (XRD) and by FTIR spectroscopy in reflectance configuration (S1 Fig 3 in S1 File) to identify substrate compounds and differentiate them from those of the paint. From XRD results, calcite (CaCO_3_) was identified as the major phase of the substrate, in addition to other mineral phases like quartz (α-SiO_2_), gypsum (CaSO_4_·2H_2_O), celestite (SrSO_4_), and lower amount hydroxyapatite Ca_5_(PO_4_)_3_(OH) and whewellite (CaC_2_O_4_⋅H_2_O). IR reflectance analyses performed on the surface of sample AC10 confirmed the expected composition, with strong signals of calcium carbonate, gypsum, and variable presence of calcium oxalates (whewellite-like), and hydrated silicates species like kaolin (S1 Fig 3B in S1 File), the latter identified in the most orangish part of the sample. SEM-EDX and FTIR/ATR analyses of the cross-section of sample AC11 identify calcite, Ca-oxalates, celestite and silicates material as main component of the orange/brownish coatings characteristic of the exposed rock surface (S1 Fig 4 in S1 File). Considering both the morphological (radial crystals features) and geochemical characteristics, this crust is likely a result of biological constructions [39]. A detailed description of the results of both samples is reported in the supporting information. Overall, the results obtained from the substrate are consistent with those of the EDXRF analyses explaining the high presence of Ca, S and Sr in the EDXRF spectra of the substrate.

### Characterization of the paintings

#### In situ non-invasive EDXRF analyses

The paintings were produced in various shades of red, ranging from red to dark purple. Visual assessment of the colors is often affected by alteration factors and, mainly, by surface patinas or deposits of varied color ranging from orange to dark brown. Interestingly, the EDXRF spectra recorded on the paintings (S1 Fig 2 and S1 Table 1 in S1 File) show that regardless of the color nuance, all red motifs presented very similar spectra, with higher amount of Fe than the substrate (Fig 5B–black line). This suggests the use of iron-based compounds, probably Fe oxides/hydroxides (e.g., red ocher, hematite, etc.), as main raw material. These results are in line with those reported in the archaeological literature on the nature of the red pigments used by Levantine artists [11,12]. No additional minor and/or trace elements were detected. As in rock surface, the randomly varying amount of S in the spectra of the red motifs is probably related to the presence of external sulphate-based patinas randomly covering the paintings. The calculated fluorescence line areas of the most significant elements (S, K, Ca, Ti, Mn, Fe, and Sr), representative of the composition of all the analyzed points, and the codes assigned to the analyzed spots are displayed in S1 Table 1 in S1 File. Plotting the normalized areas of the Fe(Kα) fluorescence lines versus those of Mn(Kα), some further indications can be outlined about the constitutive materials of the motifs (S1 Fig 5 in S1 File). The presence of Mn, beyond its detection in the rock surface (red circles in S1 Fig 5 in S1 File), seems correlated to the iron for some motifs (it is important to note that some measurements collected from the same motif show different amounts of Fe/Mn, probably due to the thickness and/or deterioration of the pictorial layer [40]). Some EDXRF spectra of the pigmented zones show a Mn content similar to unpigmented areas, with a higher/lower amount of Fe obviously due to the red pigment of the motifs (black squares in S1 Fig 5 in S1 File). For example, the spectra of motifs 6 and 7 (both in orange/reddish color) have an amount of Mn/Fe very similar to those of the substrate. While in another group of measurements the net areas of the Mn and Fe are significantly higher than in the substrate, as visible in most of the points collected in motifs 9 and 2 (of purplish color). These results could suggest i) the use of different sources and ii) that some of the iron-based raw material used to depict these motifs show Mn inclusions. Thus, EDXRF in-situ analyses could provide preliminary indications of the use of different raw materials in the different phases of art creation, as previously reported in the study of pigments from the site of la Saltadora (Castellón, Spain) [19].

#### SEM-EDX, micro-Raman and FTIR analyses of the painting cross-sections

Motivated by the search for answers to specific archaeological questions and guided by data obtained from previous non-invasive EDXRF analyses, targeted sampling of the painted motifs and areas of superimposition was carried out. The aim was to further investigate the paint composition and the stratigraphy, as well as the pigment production technologies and recipes used by Levantine and Schematic artists.

**Fig 6:**
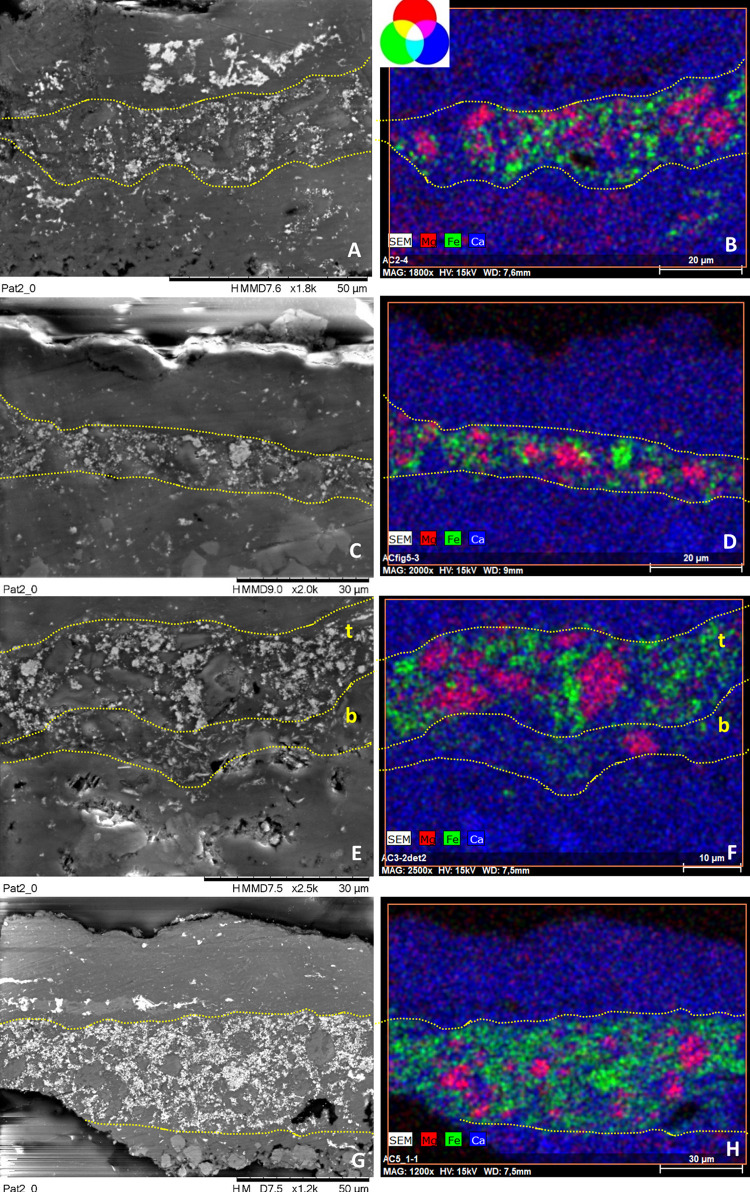
SEM backscattered images and their RGB composite images of the elemental distribution of Mg_Kα_/Fe_Kα_/Ca_Kα_ of a selected area of cross-sections AC2.1 (images A-B), AC2.2 (images C-D), AC3 (images E-F), AC5.1 (images G-H) constitutive of group 1. See S1 Figs 6 and 8 in S1 File to visualize the location of the analyzed areas within the cross-section (framed by red squares).

**Fig 7:**
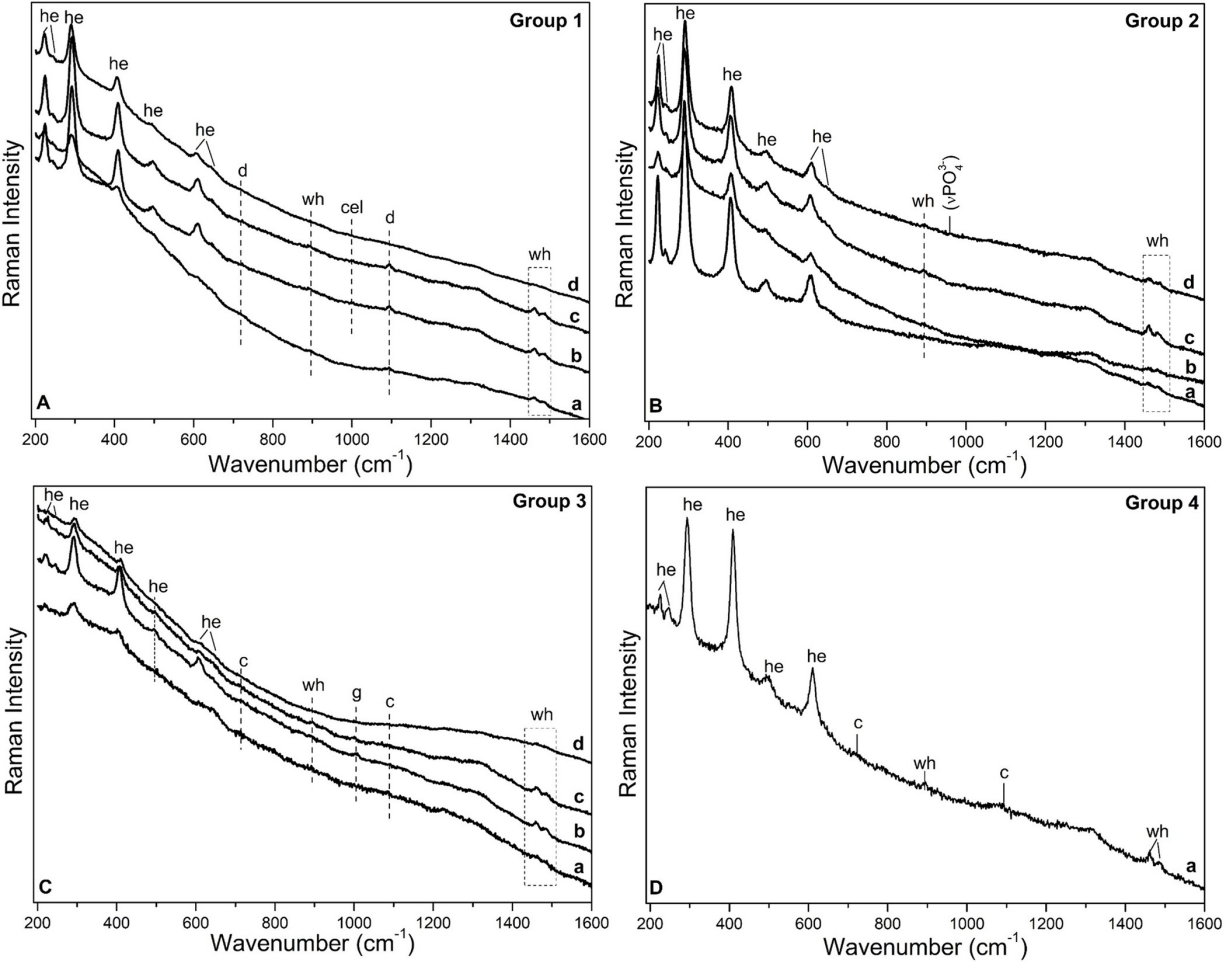
Representative Raman spectra (λ_exc_ = 785 nm) acquired in the pictorial layers of the cross-sections belonging to A) group 1 (line a = AC2.2, line b = AC2.1, line c = AC3_top_, line d = AC5.1); B) group 2 (line a = AC1_top_, line b = AC4, line c = AC5.2 and line d = AC8); C) group 3 (line a = AC1_bottom_, line b = AC3_bottom_, line c = AC6 and line d = AC9); D) group 4 (line a = AC7). Legend: he = hematite; d = dolomite; wh = whewellite; cel = celestite; c = calcite; (νPO_4_^-3^) = phosphate group assigned to apatite.

**Fig 8:**
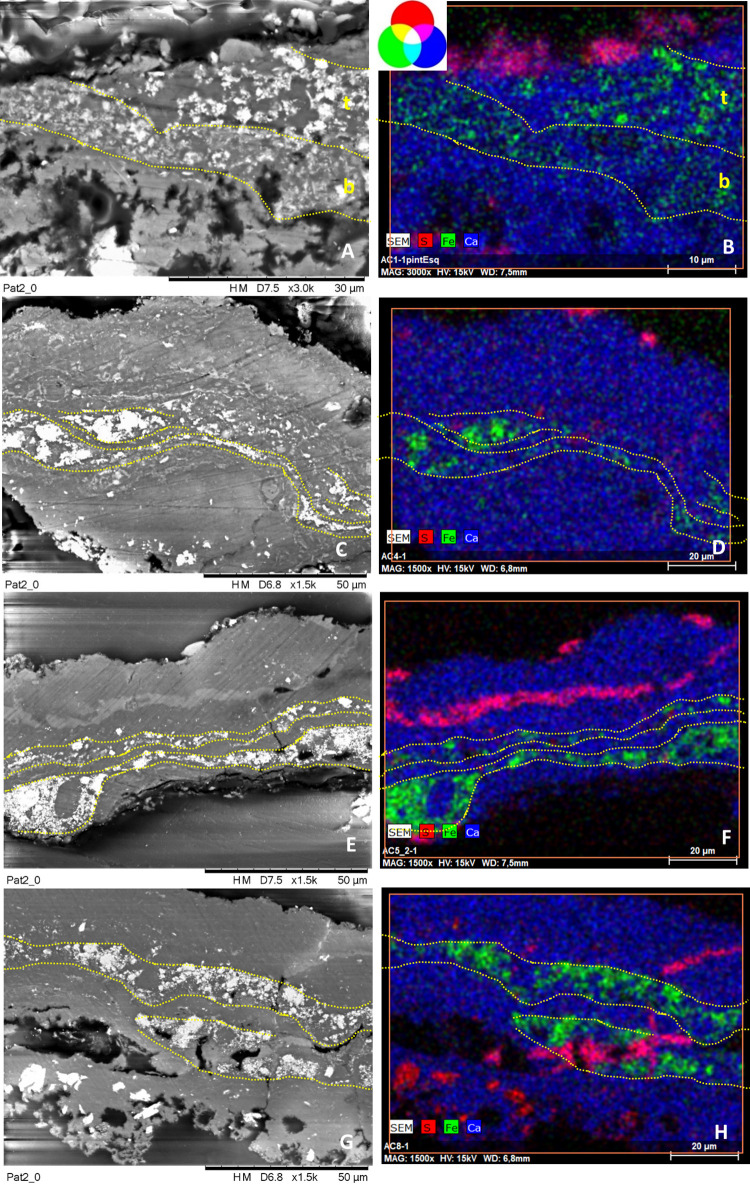
SEM backscattered images and their RGB composite images of the elemental distribution of S_Kα_/Fe_Kα_/Ca_Kα_ of a selected area of the cross-sections AC1 (images A-B), AC4 (images C-D), AC5.2 (images E-F), AC8 (images G-H) constitutive of group 2. See S1 Figs 6–8 in S1 File Figs to visualize the location of the analyzed areas (framed by red squares).

**Fig 9:**
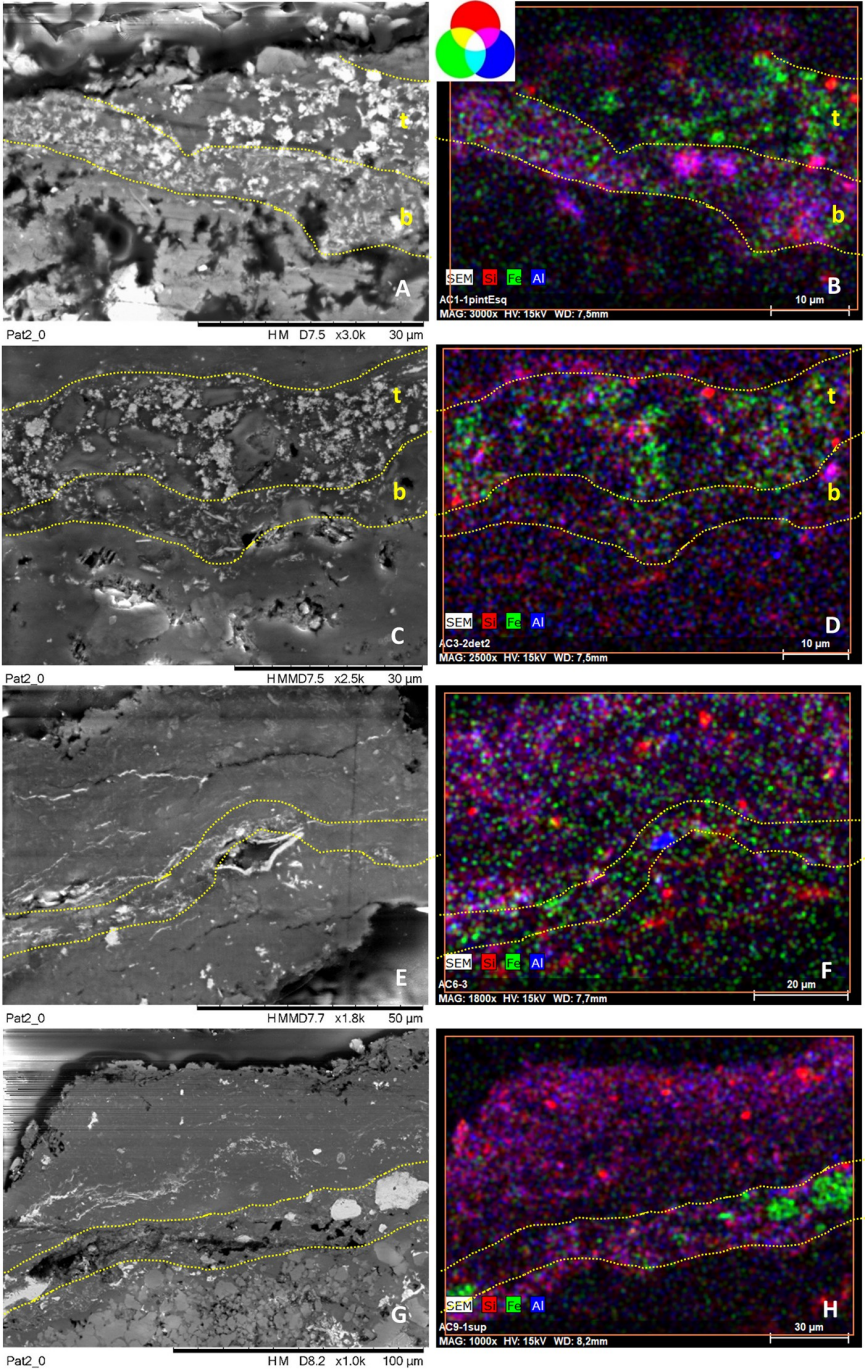
SEM backscattered images and their RGB composite images of the elemental distribution of Si_Kα_/Fe_Kα_/Al_Kα_ of a selected area of cross-sections AC1 (images A-B), AC3 (images C-D), AC6 (images E-F), AC9 (images G-H) constitutive of group 3. See S1 Figs 6–8 in S1 File to visualize the location of the analyzed areas (framed by red squares).

Sample cross-sections have been investigated by SEM-EDX, micro-Raman spectroscopy and Fourier Transform Infrared spectroscopy in reflectance configuration, after examination by Optical Microscopy. All samples show a similar microstratigraphic structure, regardless of whether they belong to a single motif or to two superimposed motifs. Cross-sections share four main features, as shown in the OM and SEM pictures (Figs 3 and 4 and S1 Figs 6–8 in S1 File). From bottom to top, the cross-sections include:

- the rock, with higher porosity as it nears the surface, where
- a dense and compact patina, of which the aspect and texture indicate a biological origin, is coating it. Calcium-oxalates in the form of whewellite (CaC_2_O_4_⋅H_2_O) and calcite (CaCO_3_) have been identified as main components of this interstratified coating, with a random presence of calcium and strontium sulphates formations, namely gypsum (CaSO_4_·2H_2_O) and celestine (SrSO_4_). Calcite is the main component of the substrate, while in some samples (AC1, AC7 and AC9) needle-like crystals of celestine are also identified in the substrate (S1 Fig 9 in S1 File). Following,
- a red pictorial layer, constituted by hematite (Fe_2_O_3_) as the main mineral phase. While hematite is the main component of the red paints, different motifs show different hematite-based formulations, as discussed below. The paint layer iii shows both thicknesses (ranging from 5 μm to 50 μm) and a number of strata variables, depending on the micro fragment analyzed. In fact, it can present either a single red block with the same composition, like in cross-sections AC2.1, AC2.2, AC5.1, AC6, AC7 and AC9; or two adjacent red pictorial strata (referred to as top and bottom in the text) with different formulations, as seen in samples AC1 and AC3; or the same composition but disrupted by interstratified layers of oxalates and/or gypsum, as in cross-sections AC4, AC5.2, and AC8. Small black grains of amorphous carbon, most likely of biological origin, are visible at the bottom edge of the painted layers (samples AC3, AC6, and AC8) and at the interface between them and the underlaying patina.
- An external compact coating/crust of variable width, stromatolite-like appearance and similar texture and shape to layer ii, evenly covers the red paint. This layer is variably composed by whewellite (CaC_2_O_4_⋅H_2_O), gypsum (CaSO_4_·2H_2_O), celestine (SrSO_4_) and silicates formations, as clayish minerals. The latter are probably related to aeolian materials, such as dust adhering to the surface.

Similar microstratigraphic structure and constitutive materials have been also identified in other analysis of Levantine rock art micro-samples [11].

Regarding the **composition and characterization of the red pictorial layers**, as already mentioned, all of them are hematite based. Hematite and/or iron-rich compounds like red natural ochres [41] are the most common minerals used in rock art paintings around the world, Levantine and Schematic art included [1–4,11]. Through the results of the SEM-EDX and micro-Raman analyses of the cross-sections we have identified four different pictorial recipes or paint mixtures (group 1–4), all with their own characteristics in both elemental compositions and vibrational profiles (Table 2). Whether or not the mineral mixtures identified are natural or intentionally made by the artists is not yet proven. Other researchers have already discussed that different paint mixtures could either represent different paint recipes, or natural formations from similar or different raw material sources [42].

**Table 2:** Results of the analyses of the pictorial layers of cross-sections AC1-AC9, divided into four groups according to the paint mixtures identified. The percentual amount of the elements within brackets are < 1%. Quantitative micro-analyses are reported in S1 Table 2 in S1 File. *for sample AC4 and AC8 the results of the top layer are reported. (sup. = superimposition).

Paint Layer Characterization				
Groups	Sample and Motif n^o^	Elemental Composition	Fe (w%)	Main Phases Identification
**Group 1**	AC2.1 –sup. 5/6 AC2.2 –sup. 5/6 AC3_top_−sup. 5/6 AC5.1 –sup. 10/11	O, C, Ca, Fe, Mg (Si, Al, Cl, K, S, Na, P) O, C, Ca, Fe, Mg (Si, Al, Na, S, Cl, K, P) O, C, Ca, Fe, Mg, Si (S, Al, K, Cl, Na, P) O, C, Ca, Fe, Mg (Si, Al, S, K, Sr, Cl, Na, P)	9,7% 8,2% 11,7% 13,2%	Hematite (size < 1 μm) + dolomite + whewellite
**Group 2**	AC1_top_−sup. 6/7/9 AC4*–sup. 10/11 AC5.2 –sup. fig. 10/11 AC8*–Fig 2	O, C, Fe, Ca, Si (Al, K, Mg, S, Cl, Na, P, Mn O, C, Fe, Ca (Si, Al, K, Mg, S, Na, Sr, Cl, P) O, C, Fe, Ca (Si, S, K, Mg, Al, Na, Cl, P) O, C, Fe, Ca (Si, Mn, Mg, Al, Na, S, Cl, P)	15,5% 15,9% 22,1% 20,9%	Hematite (size < 1 μm) + whewellite
**Group 3**	AC1_bottom_−sup. 6/7/9 AC3_bottom_−sup. 5/6 AC6 –fig 13 AC9 –fig 3	O, C, Ca, Fe, Si, Al, K (Mg, P, S, Cl, Na) O, C, Ca, Fe, Si, S (Mg, Al, Na, K, Cl, P) O, C, Ca, Fe, Sr, Si, S (Al, Mg, P, Cl, K) O, C, Ca, Fe, Si, Al (S, Mg, K, P, Cl, Na)	8,4% 8,6% 3,7% 7,2%	Hematite (size < 1 μm) + clay matrix + whewellite
**Group 4**	AC7 –Fig 4	O, C, Fe, Ca, Si (Al, K, Mg, S, Cl, P, Na)	14,3%	Elongated hematite + whewellite

#### The first group (group 1) includes the paint layers of cross-sections AC2.1, AC2.2, AC5.1 and the top stratum of sample AC3, referred to as AC3_top_ (Figs 3 and 4)

SEM-EDX analyses of the pictorial stratum of these cross-sections (Fig 6) show they are very compact and composed by an important amount of Fe-rich pseudospherical crystals (in green in the SEM-EDX elemental mapping of Fig 5B, 5D, 5F and 5H), with sizes ranging between 100 to 300 nm, charged with polygonal crystals based on Ca and Mg of size ca. 10 μm. These latter are well correlated as displayed in magenta in the RGB composite images created by the superimposition of the elemental mapping of Mg_Kα_/Fe_Kα_/Ca_Kα_ for the red/green/blue channels in Fig 6B, 6D, 6F and 6H, suggesting the use of dolomite (CaMg)CO_3_ together with the iron-oxide. The paint layer thickness is variable and ranges from 10 to 50 μm, depending on the sample. All the cross-sections include the substrate.

**Fig 10:**
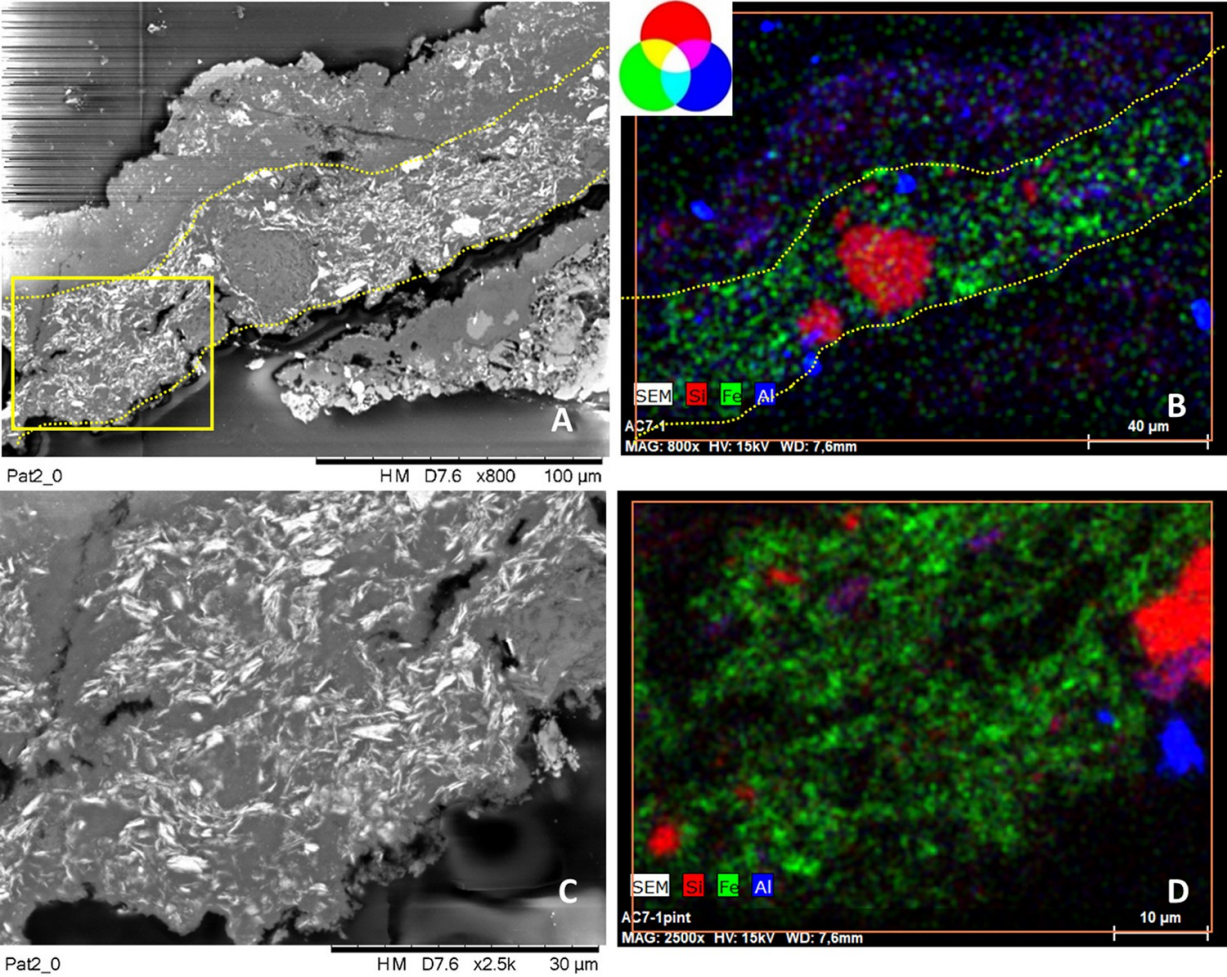
A, C) SEM backscattered images and its zoomed area (yellow square in A), and B, D) their RGB composite images of the elemental distribution of Si_Kα_/Fe_Kα_/Al_Kα_ of a selected area of cross-sections AC7 constitutive of group 4. See S1 Fig 8 in S1 File–top image to visualize the location of the analyzed area (framed by red square).

**Fig 11:**
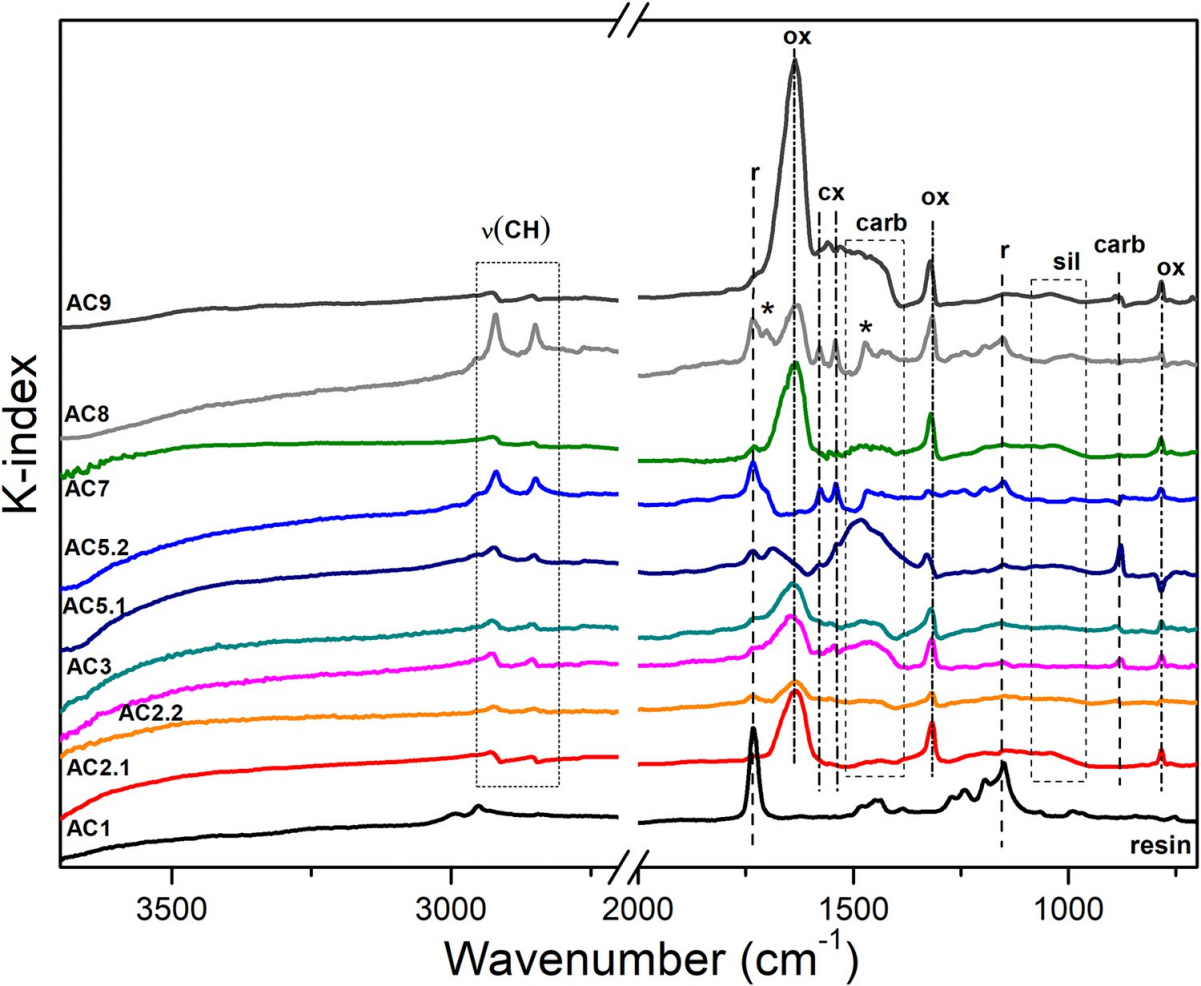
Representative FTIR spectra (kk transformed) extracted from the pictorial layers of each investigated areas of the examined cross-sections. Legend: r = resin; ox = calcium oxalates; carb = calcium carboxylates; sil = silicate; the asterisk * refers to the signals of a free fatty acid species [58].

Focusing on samples AC2 (divided into two fragments AC2.1 and 2.2) and AC3, they both belong to the superimpositions of motifs 5 and 6, but the presence of two distinct layers is only visible in sample AC3 (S1 Fig 10 in S1 File). These two layers (top and bottom in AC3 cross-section, Fig 6E and 6F) are perfectly superimposed and no intermediate stratum occurs between them. The top layer is constituted by hematite+dolomite and belongs to group 1. Instead, the bottom layer is more orange and will be discussed as part of group 3. As sample AC2, cross-section AC5.1 only shows one red stratum, even though it was collected at the overlapping area between motifs 10 and 11. In group 1, the weight percentage of Fe in the red layers ranges between 9–13% and that of Mg between 2–4% (Table 2 and S1 Table 2 in S1 File). Lower amounts of Al, Si and K are also randomly spread in the paint layers (Table 2 and S1 Table 2 in S1 File, and S1 Fig 11B, 11E, 11H and 11K in S1 File) while gypsum and celestite formations occur along the cross-sections, as shown in magenta and yellow respectively in the RGB composite images created by the superimposition of the elemental mapping of S_Kα_/Sr_Lα_/Ca_Kα_ for the red/green/blue channels in S1 Fig 11C, 11F, 11I and 11L in S1 File.

Micro-Raman investigations confirm hematite as the unique iron oxide phase constituting the red painting layers in the cross-sections (see S1 Tables 3 and 4 in S1 File). As main features, the seven characteristic vibrational modes of hematite located at 221–225 (A1g), 243–245 cm^-1^ (Eg), 288–294 cm^-1^ (Eg), 405–410 cm^-1^ (Eg), 493–498 cm^-1^ (A1g), 604–610 cm^-1^ (Eg) and 1315 cm^-1^ -this latter is only detected using 532 nm laser excitation and it is assigned to a two-magnon scattering of hematite- are identified in most of the samples as main constitutive component of the red layers [36,43]. Moreover, all the red layer spectra show a weak band, like a shoulder, at ca. 645–660 cm^-1^. The assignment of this band is still under debate since it could be attributed to any residual presence of magnetite in the hematite compound [36], or it could be due to the hematite itself, due to disorder effects in the crystal lattice [44,45]. Moreover, disordered hematite can originate either in nature due to natural defecting, Al substitution, weathering or due to anthropic actions such as grinding, heating procedures, etc. [46–50]. In fact, it is well-known that, under heating or grinding (so far only demonstrated with industrial mills) [49,50], goethite dehydrates forming hematite and this argument is of great interest in archeological contexts. At this stage, we cannot exclude any of these options regarding the assignment of the band at 645–660 cm^-1^. In all paint spectra the monohydrate form of Ca-oxalate, whewellite, (CaC_2_O_4_⋅H_2_O), has been identified, characterized by the intense Raman doublet at 1458-62/1484-88 cm^-1^ assigned to the C = O symmetric stretching mode of whewellite (ν_s_C = O). The 1631 cm^-1^ peak is related to antisymmetric C = O stretching (ν_a_C = O), while the band at 895 cm^-1^ is due to the C-C stretching mode (νC-C). Finally, the bands at 137 and 190 cm^-1^ are ascribable to the lattice modes (S1 Tables 3 and 4 in S1 File) [51–54].

While hematite is always present, the Raman spectra of the red paints of the different samples show several differences both in terms of relative intensity and bandwidth of the iron oxide peaks and in composition. In group 1 (Fig 7A, S1 Tables 3 and 4 in S1 File), paint layers are characterized by the use of hematite and dolomite, CaMg(CO_3_)_2_, confirming SEM-EDX observations. Dolomite is identifiable by the peaks at 1096–1093 (Ag), 722 (Eg) (very weak) and 173–176 (Eg) cm^-1^ which are due to the symmetric stretching (ν_1_), bending (ν_4_), and external vibrations of carbonate group (CO_3_^-2^) of dolomite, respectively (S1 Tables 3 and 4 in S1 File) [55,56]. Motifs in this first group are purple.

#### Group 2 includes the red paint layers of samples AC4, AC5.2, AC8, and the top stratum of sample AC1, named AC1_top_ (Figs 3 and 4, Table 2)

**Fig 12:**
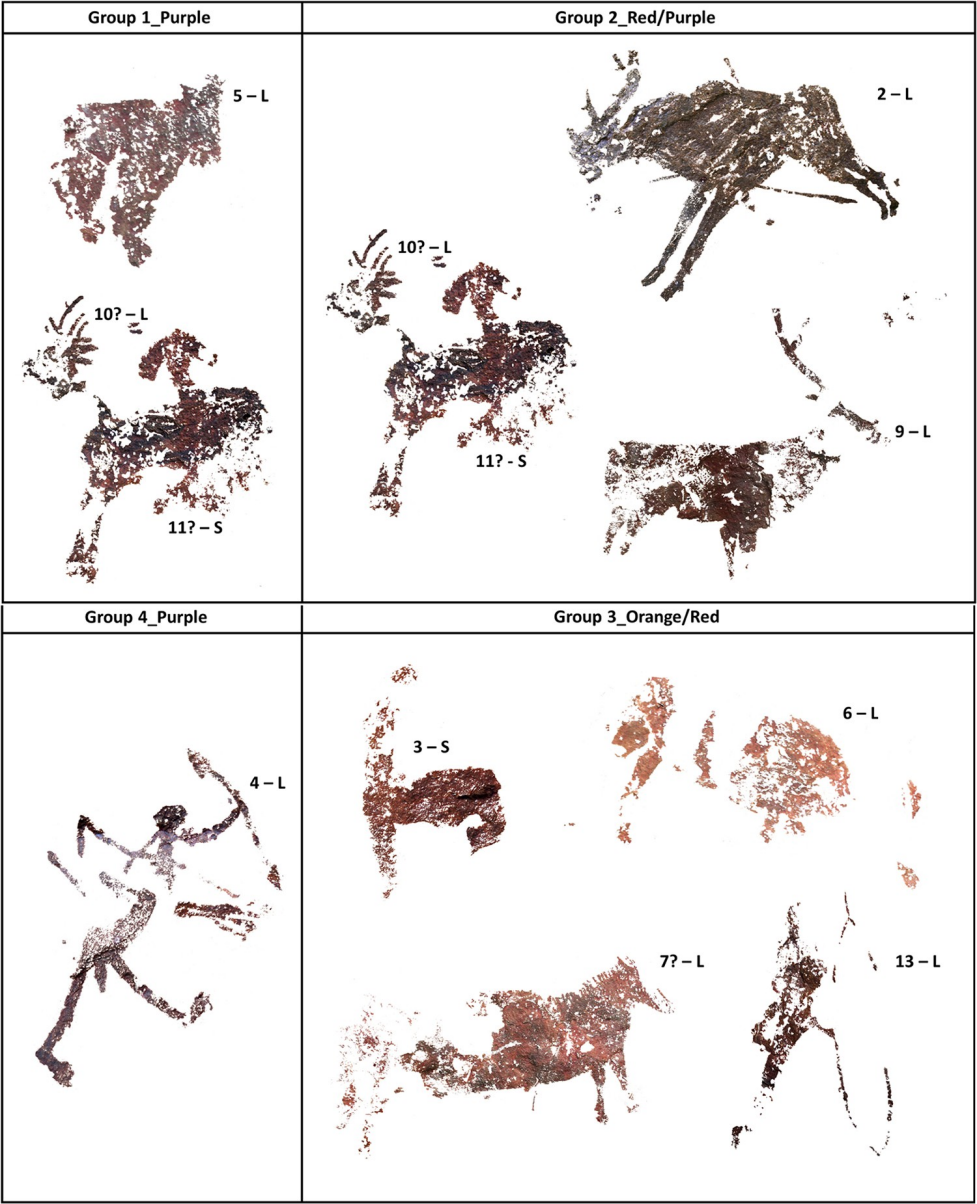
Motifs corresponding to each group. L and S refer to Levantine and Schematic styles, respectively.

SEM-EDX analyses (Fig 8) show that the pictorial layers of these samples are made up of Fe-rich particles of submicron crystals (size < 1 μm), attributable to iron oxides, (in green in the SEM-EDX elemental mapping of Fig 8B, 8D, 8F and 8H), with sizes ranging between 50 and 300 nm, occasionally forming clusters up to ca. 10 μm. The Fe-rich grains are included within a calcium-based matrix (Table 2 and S1 Table 2 in S1 File). These layers are the richest in iron among the four groups, whose weight percentage ranges from 15–22% (Table 2 and S1 Table 2 in S1 File). The red layers have no significant amount of characteristic elements (Al, K and Si are all <1%—S1 Table 2 and S1 Fig 12 in S1 File), nor do they contain Mg as in the previous group. In line with the in situ XRF analyses, the pictorial layers of cross-sections AC8 and AC1_top_, belonging to motif 2 and to the superimposition of motifs 6, 7 and 9 respectively, also display a low amount of Mn, as shown in the mapping of S1 Fig 13 in S1 File. This evidence also might suggest that the top layer of sample AC1 belongs to motif 9.

The thickness of the paint layer of the cross-sections ranges from 10 to 20 μm, depending on each sample. In sample AC1, only two of the three expected strata are distinguished. They have different composition and texture and, as sample AC3, they are perfectly adjacent to each other. On the contrary, in samples AC4, AC5.2 and AC8 the paint layer does not appear as a single block, but a dense parallel layer rich in calcium (composed by whewellite as identified by micro-Raman analyses) is present as if dividing the paint layer into two strata, whose composition is similar according to SEM-EDX microanalyses (Fig 8, Table 2 and S1 Table 2 in S1 File). In particular, samples AC4 and AC5.2 belong to the superimpositions of motifs 10 and 11, while AC8 belongs to motif 2. In all three cases, the microstratigraphy of the fragments does not contain the substrate. Their composition and texture are very similar, both within and between samples. Moreover, well-defined gypsum rich parallel levels (in magenta in the RGB composite images created by the superimposition of the elemental mapping of S_Kα_/Fe_Kα_/Ca_Kα_ for the red/green/blue channels shown in Fig 8) are also present in these samples and, in cross-section AC8, they also interfere with the pictorial layer (Fig 8H). Defined levels rich on P are also distinguishable, distributed in the calcium-rich strata (S1 Fig 12C, 12F, 12I, 12L in S1 File). Raman analyses highlight the presence of calcium phosphate, as apatite (as will be further discussed below). Considering their stratigraphic distribution, these phosphates layers could be bioconstructions [57]. Micro-Raman spectra of group 2 (Fig 7B, S1 Tables 3 and 4 in S1 File) show that the peaks of hematite are all quite intense and narrow, presenting better scattering as well among all the investigated groups. Whewellite signals are also detected within the paint layers. Moreover, in some spectra of the paint layers of cross-sections AC5.2 and AC8 signal at 1445 cm^-1^ has been detected. This band has been assigned to a calcium carboxylate species [58]. Further discussion will be addressed later in the FTIR results section. Confirming SEM-EDX results, in samples AC4, AC5.2 and AC8, with the pictorial layer divided into two levels, no spectral differences between both layers have been identified. High whewellite signals have also been identified both in the Ca-based interstratified layers dividing the paint strata and within these latter. Motifs in this second group are red/purple.

#### Group 3 includes the red paint strata of samples AC6 and AC9 and the bottom layers of AC1 and AC3, named AC1_bottom_ and AC3_bottom_ respectively (Figs 3 and 4)

Based on SEM-EDX analyses (Fig 9 and S1 Fig 14 in S1 File), they are composed of very tiny Fe particles within Al, Si and K matrix assigned to clay minerals, also identified by the flat morphology of their crystals. The former appears in green in the RGB composite images of the elemental mapping of Si_Kα_/Fe_Kα_/Al_Kα_ for the red/green/blue channels. The RGB composite figures of the elemental maps of Al_Kα_/Si_Kα_/K_Kα_ are shown in S1 Figs 11G, 11H, 12A, 12B, 14B and 14E in S1 File. The weight percentage of Fe in samples of group 3 ranges between 4–8% (Table 2 and S1 Table 2 in S1 File) and it is the lower among the four identified groups. Unlike the other groups, the painting layers of this group are highly heterogeneous: for example, the pictorial layer of cross-sections AC6 and AC9 appear highly diluted in some points while in others iron (III) oxide crystals of larger size, up to 50 microns, are present (and appear as black agglomerates under the optical microscope, and displayed in Fig 4H and 4K). In these samples, defined levels of gypsum and celestite interfere also with the pictorial layers (S1 Fig 14C, 14F in S1 File). The micro-Raman spectra (Fig 7C and S1 Tables 3 and 4 in S1 File) of group 3 show very broad and not very intense hematite peaks, which can be related to the low crystallinity and the disordered structure of the investigated material. Moreover, a very pronounced fluorescence background affected the micro-Raman investigations, which is in accordance with the presence of clay matrix in the pictorial layer [59]. Again, signals of whewellite are detected in the investigated paint layers. If we focus on samples AC1_bottom_ and AC3_bottom_, collected from the superimpositions of motifs 6, 7, and 9 and 5 and 6 respectively, they have in common motif 6, the orange-like zoomorphic motifs. In both samples, the bottommost stratum laying directly on the coated substrate shows similar features both at SEM-EDX and micro-Raman. In addition, considering the OM pictures of sample AC3 (S1 Fig 10 in S1 File), an orangish layer is recognized in the central part of the cross-section (area where both SEM-EDX and Raman analyses were performed) suggesting that the motif underneath is the 6. The hue of the motifs belonging to group 3 is red/orange.

Finally, **Group 4 includes only the pictorial layer of sample AC7, collected from motif 4** (Fig 4). SEM-EDX analyses (Fig 10 and S1 Fig 15 in S1 File, Table 2 and S1 Table 2 in S1 File) show that the paint is constituted by Fe-rich crystals displaying a well-defined acicular morphology characterized by ca. 3 μm in length and 100–300 nm in thickness (Fig 10A and 10C). Such elongated shape of the hematite particles differs from all the others paint samples. The weight percentage of iron (wt%) in this sample is about 14% (Table 2 and S1 Table 2 in S1 File). Microanalyses also display high amounts of Ca as well as sparse crystals composed by Si, K and Al (S1 Fig 15 in S1 File). Hematite peaks appear very intense and narrow in the Raman spectra (Fig 7D). The main bands at 294 and 412 cm^-1^ show equivalent high relative intensities while the band at 222 cm^-1^ appears quite weak compared to the other. This spectral behavior is different to the Raman curves of the other samples. Whewellite is also detected within the paint layer. The color of motif 4 is purple.

Regardless of the specificity of the four groups, other components are also randomly detected in the investigated red layers. Focusing on micro-Raman investigations (S1 Tables 3 and 4 in S1 File), peaks at 200–205, 260–264, 350–355, 464, 1160 cm^-1^ can be attributed to α-quartz (α-SiO_2_), while the bands at ca. 260, 270, 445, 480 and 510 cm^-1^ could be due to other aluminosilicates minerals, like species belonging to feldspar group [60–62]. Likewise, weak bands located at 960, 998–1001, 1006–1007 and 1085–1088 cm^-1^ are sometimes identified. They are assigned to the symmetric stretching mode of the tetrahedral phosphate anion ν_1_(PO_4_^3-^) of apatite, Ca_5_(PO_4_)_3_(F, Cl, OH), and symmetric stretching mode (ν_1_) of the S–O bond in the sulfates due to the presence of celestite (SrSO_4_) and gypsum (CaSO_4_·2H_2_O), and to the symmetric stretching (ν_1_) mode of carbonate group (CO_3_^-2^) of calcite (CaCO_3_), respectively [63–67]. The identification of these compounds amply justifies the results of the SEM-EDX mapping, in which S + Ca and S + Sr are highly correlated with each other along the analyzed cross-sections as well as the identification of P rich layers. Furthermore, the presence of amorphous carbon in correspondence with some black spots in the interface between the pictorial layer and the underlying calcium oxalate coating in samples AC3_bottom_, AC6 and AC8, is confirmed by Raman. The typical D1 and G bands of amorphous carbon at 1319 and 1590 cm^-1^ are detected, even they appear very weak and broadened. This presence can be referred to as biological origin [25,68]. Apart from the pictorial layers, signals of calcite (CaCO_3_) with its typical bands located at 1084, 711, 278, and 154 cm^-1^, that are given to the symmetric stretching (ν_1_), symmetric stretching deformation/bending (ν_4_), and external vibrations of carbonate group (CO_3_^-2^) [66] have been identified on the substrate, as expected by its XRD analysis, while a very intense fluorescent signal interfered when the external coatings (as previously mentioned as iv) have been analyzed. This is a recurrent problem when analyzing these kind of samples by micro-Raman spectroscopy [69,70].

Finally, FTIR analyses in reflectance configuration were used to identify **the presence of binders** (see Fig 11).

However, we faced difficulties in recognizing any specific signals related to any residual binders. In fact, besides the identification of the CH stretching at ca. 2900–2800 cm^−1^, that could be related to any lipidic material [71], several signals have hindered the possibility to detect any peculiar peaks characteristics of a remaining binding media such as lipid-based medium or proteinaceous binders [72,73], most likely binders used by prehistoric painters [11,12]. First, the residual resin signals at 1730 cm^-1^ and 1500–1410 cm^-1^. Its presence and penetration in the sample is related to the embedding procedures. This problem is accentuated when porous samples, such as this, are managed [74]. Second, the strong antisymmetric stretching mode of calcium-oxalates at ca. 1640 cm^-1^ (ν_a_(CO)) identified in all the pictorial layers [38]. And third, the wide asymmetric stretching of carbonate band (ν_3_) centered at ca. 1440 cm^-1^ [71].

Moreover, bands attributed to silicate are also detected in the pictorial layers of cross-section AC1, AC2.1, AC5.1, AC7 and AC9. However, in microsamples AC1, AC2.2, AC5.1, AC5.2 and AC8 two additional narrow peaks at ca. 1580 and 1540 cm^-1^ are also detected in the pictorial layers (such signals also occurred randomly in layer ii and iv of those cross-sections). Considering both the shape and the position of these bands, these signals could be attributed to the presence of calcium monocarboxylic fatty acid salts [75]. In fact, the strong doublet at 1580 and 1540 cm^−1^ corresponds to the asymmetric stretching bands of the COO− moiety. Metal carboxylates are widespread alteration products commonly related to pictorial art that are due to the degradation of certain pictorial binders (such as siccative oils, proteinaceous materials, natural resins, etc.) and their interactions with metallic ions present in the pictorial layer/substrate. Their formation and identification are well documented when analyzing easel paintings [76], but, based on our knowledge, it would be the first time they are identified in rock art. Considering that carboxylate signals were not identified on substrate samples AC10 (S1 Fig 4B in S1 File) and AC 11 (S1 Fig 5B in S1 File), their identification in the paint microsamples could be related to the degradation of a binder. Similarly, when the presence of oxalates is detected in the pictorial layer it could be also related to the degradation of binders, as further considered in the discussion section. However, this hypothesis about the presence of Ca-carboxylates must be further investigated to avoid misinterpreting the results. It is not to exclude that metal carboxylates could have been also formed by other modern contaminants, such as:

- any undocumented past treatment of the painted motifs (for example, twenty years ago at Val del Charco del Agua Amarga site (Alcañiz, Teruel) the use of paraffin wax as a procedure to enhance motifs visualization was reported) [77]. Whether or not the use of this or similar materials became widespread beyond this site is not known, even though it is not reported in the scientific literature;
- any possible interaction between the embedding resin used in the analysis, a polymethyl methacrylate-based medium, and the metallic calcium ions present in the layers [78];
- the interaction with the lipid components of the human epidermis (e.g., accidental contact of the microsamples/cross-sections with the fingers).

Finally, beyond the paint layers, the FTIR analyses showed also signals of calcium-oxalates, whewellite like, are the main constituents of the external (iv) and second layer (ii) of the cross-sections, referred to as iv and ii respectively at the beginning of the results section. They show bands at 1640, 1320, and 785 cm^-1^ that are assigned to the CO antisymmetric and symmetric stretching mode of the oxalate anion, ν_a_(CO) and ν_s_(CO), and the bending mode δ(OCO) of whewellite, respectively [38]. Moreover, the asymmetric stretching (ν_3_) of carbonate group (CO_3_^-2^) as broad band centered at 1440 cm^-1^ is also visible in layer i and ii, together with the bands at 880 cm^-1^ and 715 cm^-1^, due to the out-of-plane bending (ν_2_) and to in plane-bending (ν_4_) characteristic of calcite [66].

## Discussion

The analyses performed in the Levantine and Schematic paintings of el Carche rock art site, both in situ and in micro-samples, offer new insight into the materials, the potential and challenges of microstratigraphic analysis (including superimpositions and crusts), and the peculiarities of the different styles documented in panel.

Of particular interest is the identification of four different paint compositions:

- Paint from group 1, identified in samples AC2.1, AC2.2, AC3_top_, and AC5.1, consists of hematite with submicrometric size charged with dolomite, and sporadic presence of aluminosilicates.
- The second group (samples AC1_top_, AC4, AC5.2 and AC8) shows the use of submicrometric hematite alone, with the occasional presence of aluminosilicates and impurities of Mn.
- The third (samples AC1_bottom_, AC3_bottom_, AC6 and AC9) shows the use of submicrometric hematite within clay matrix.
- The last group, with a single sample (AC7) exhibits elongated hematite occasionally charged with aluminosilicate compounds.

### Analysis of the superimpositions

The stratigraphic analysis of the polished cross-sections has partially contributed to sequence the superimpositions, which was one of the main goals of this paper. Unfortunately, not all samples collected in areas of overlapping motifs preserve the expected number of pictorial layers (e. g. samples AC1, AC2.1, AC2.2, AC5.1 and AC5.2). Sometimes, as in samples AC3, the layers are only partially visible. In this case, the bottom layer is particularly thin and only visible in some parts of the cross-section (S1 Fig 10 in S1 File). This suggests that some of the rock paintings were probably partially lost or damaged by the time new motifs were added. This is interesting as it shows deterioration processes took place already in prehistoric times. If we focus on samples AC1, AC2 and AC3, in theory related to overlapping motifs 6, 7 and 9 (AC1) and motifs 5 and 6 (AC2 and AC3) respectively, all of them have motif 6 in common and they preserve part of the rock in the stratigraphic cross-section. However, sample AC1 only preserves two of the three paint layers expected; cross-sections AC2.1 and AC2.2 only preserves one while AC3 preserves both. Thus, this later sample has become our benchmark within this group. Our analytical results show that the orange-like zoomorphic motif 6 is the lower and the purple human motif 5 lies on top of it. Chemical analyses have shown that the lower layers of AC1 and AC3 (AC1_bottom_ and AC3_bottom_) have similar compositions, and both have been assigned to group 3. Furthermore, observing sample AC3 under the optical microscope a lower layer of orangish color is distinguished, a hue that matches that of motif 6 (S1 Fig 10 in S1 File). Therefore, we state that red/orange Levantine motif 6 is the lowest in both cross-sections, AC1 and AC3. Samples AC2.1 and AC2.2 are homogeneously composed of hematite+dolomite and they don’t show any interstratified layers. This composition is similar to that of cross-section AC3 top layer (AC3_top_), and, from the analyses, they both belong to group 1. Consequently, motif 5 lies on top of Fig 6 and thus, it is more recent.

In identifying whether AC1_top_ layer belongs to Levantine horse 7 or purple deer 9, both a visual assessment and the analytical results obtained must be considered. The stratum is composed of small hematite particles in a dense calcium-based matrix caused by calcium oxalates, mainly composed of whewellite. This layer belongs to the recipes of group 2, consisting of purple motifs. Moreover, considering i) the presence of Mn shown both by in-situ XRF analyses of motif 9 and the microanalyses of AC1_top_, ii) the hue of both motifs, we could attribute the top layer of sample AC1 to purple deer known as motif 9. Finally, considering the more reddish hue of the horse named motif 7, we assign it to group 3. Furthermore, it is interesting to note that in both AC1 and AC3 samples the overlapped layers are adjacent to each other and are not separated by any Ca-rich biogenic patina. While this absence could be related to the time gap between the execution of the motifs (implying a short or no time gap), as already proposed for other similarly superimposed paintings in Australia [79], the truth is that the exact time interval between the painting events is unknown, since the development of a biogenic layer strongly depends (also) on the microlocal conditions and geometry. Thus, the lack of a patina doesn’t necessarily mean that there was no time gap between both interventions.

Regarding the overlap between motifs 10 (the Levantine deer) and 11 (the Schematic anthropomorphic motif), corresponding to cross-sections AC4, AC5.1 and AC5.2, we still have doubts about the order of the overlap [27]. None of the three cross-sections preserved a complete microstratigraphy, and two of them are even missing the substrate. However, we have identified at least two different compositions, belonging to two (motifs 10 and 11) or even three motifs (considering the superimposition of dark lines of different colour on top of motifs 10 and 11). Specifically, the paint layer of sample AC4 is composed of submicrometric hematite. It displays both morphological and chemical similarities with the red stratum of sample AC5.2 and, in fact, they both belong to group 2. Moreover, their pictorial layers are divided into two levels interstratified by calcium oxalates strata, showing similar compositions. On the contrary, sample AC5.1 is composed of hematite + dolomite (group 1) and in this case, the red stratum is a single block that lays above the coated substrate. Its features highly resemble those of samples AC2 and AC3_top_, already assigned to Levantine motif 5. However, the data obtained so far are not enough to clarify the sequence and clearly assign the results to any specific motif. Thus, further research efforts would be needed to clarify the sequence (e.g., studying the microstratigraphy of other fragments not yet embedded belonging to sample AC5; if necessary, planning additional sampling to each painted figure outside the overlapping areas, etc.).

The complete summary of the results is included in Table 3 and Fig 12.

**Table 3:** Summary of the results achieved by SEM-EDX, micro-Raman and FTIR analyses of the paint layers constituting cross-sections AC1-AC9. Label: SuperImp = superimpositions; LRA = Levantine Rock Art; SRA = Schematic Rock Art; cla = clay matrix; hem = hematite; dol = dolomite; whe = whewellite; Mn = Manganese impurities; (Al-S) = aluminosilicates; the brackets refer to the low amount identified. The results between brackets are occasionally detected. In samples marked with **, the order of superimpositions among motifs 10 and 11 and the composition of the corresponding single motifs have not been clarified.

Name	Order of SuperImp	Motif N° and Hue	Style	Pictorial layers	Group	Comments
**AC1**	Top/Middle Bottom	9, purple 6, orange	LRA LRA	T/M: Hem (size < 1 μm, Mn) + Whe (Al-S) B: Cla + Hem (size < 1 μm) + Whe	2 3	Only two pictorial layers are present
**AC2.1**	Top -	5, purple -	LRA -	Hem + Dol (size < 1 μm) + Whe (Al-S) -	1 -	Only one pictorial layer is present
**AC2.2**	Top -	5, purple -	LRA -	Hem + Dol (size < 1 μm) + Whe (Al-S) -	1 -	Only one pictorial layer is present
**AC3**	Top Bottom	5, purple 6, orange	LRA LRA	T: Hem (size < 1 μm) + Dol + Whe (Al-S) B: Cla + Hem (size < 1 μm) + Whe	1 3	- -
**AC4**	**	**	**	Hem (size < 1 μm) + Whe (Al-S)	2	Two pictorial layers with similar composition. The sample doesn’t preserve part of the substrate
**AC5.1**	**	**	**	Hem (size < 1 μm) + Dol + Whe (Al-S)	1	Only one pictorial layer is present
**AC5.2**	**	**	**	Hem (size < 1 μm) + Whe (Al-S)	2	Two pictorial layers with similar composition. The sample doesn’t preserve part of the substrate
**AC6**	-	13, red	LRA	Cla + Hem (size < 1 μm) + Whe	3	-
**AC7**	-	4, purple	LRA	Acicular Hem + Whe (Al-S)	4	-
**AC8**	-	2, purple	LRA	Hem (size < 1 μm, Mn) + Whe (Al-S)	2	Two layers with similar composition are present, even if the sample belongs to a single figure. The sample is unprovided of the substrate
**AC9**	-	3, purple/red	SRA	Cla + Hem (size < 1 μm) + Whe	3	-

### Materials, sources, recipes and colours

As mentioned at the beginning of the discussion section, the analysis performed show the identification of four different paint mixtures with specific compositions (Table 3 and Fig 12). From these results we can note: a richness in the choice of the orange/red/purple raw materials used by prehistoric artists to depict their motifs in this rock shelter, as well as a lack of a direct correlation between the different pictorial mixtures and the different prehistoric styles. In particular, while all the paintings in red/orange shades fit in the same group, those in darker red/purple belong to different groups, showing more variability in the paint mixtures or technologies used for this specific hue (Fig 12). This suggests that artists could have been using the same source to collect the red/orange raw materials, while different sources or different paint recipes were used for the creation of the purple motifs. In some cases, such sources remained unchanged over time (considering that both Levantine and Schematic motifs share at least two groups of paint mixtures). Whether or not this indicates short-range supply sources would have to be assessed in the future, through geological surveys. Similar results have been also found by Mas et al. [25] when analyzing two different samples of Levantine and Schematic paintings from Abrigo Grande de Minateda and Abrigo del Barranco de la Mortaja (Albacete). In that publication researchers detected comparable compositions in the two micro fragments, concluding the similarity in the composition of the paint samples between these two distinct traditions.

Unlike that study, the results of our analysis show that both Levantine and Schematic artists used several colours, raw material sources or paint recipes and paint technologies. Levantine paintings fall into the four groups identified, while Schematic art falls into two groups. This variety in the composition of paints, along with the diversity of styles recorded at this site, suggest a recurrent and extended use of the site though several phases, and therefore that this place remained culturally significant over time.

To our surprise, this variety of recipes is documented even among motifs that, based on the apparent visual similarities of the colour hues, we had considered synchronous [28]. This is the case of the best-known scene of the site, interpreted as capturing the final stage of a deer hunt. The animal (motif 2) is wounded by several spears and is either bleeding or vomiting (as suggested by a series of dots surrounding it), while the hunter (motif 4) turns the back on the prey, raising one of the arrows as a sign of success. Now, our paint analysis suggests that both figures were made with different pigments from different sources (Hem + Whe for motif 2 and Acicular Hem + Whe for motif 4), further complicating the interpretation of the scene and opening new questions that we would not have considered without the analyses performed. Whether the differences in paint composition suggest that these two motifs were produced by different artists or the same artists using different sources is difficult to tell. But considering the patterns of composition of other Levantine sites, the addition and integration of old figures into new scenes adding linear motifs is common in the final stages of Levantine art [80,81].

While hematite is the main component in all the recipes identified, morphological and spectral differences suggest the use of different sources or perhaps different paint mixtures and maybe different processing techniques. In most of the paint groups, submicrometer hematite has been identified, presenting fine granular size less than 1 μm. Similar finely ground hematite was already found at Hoz de Vicente and Sierra de las Cuerdas paintings (Cuenca) [16,17]. The authors suggested that such tiny size of the hematite was an indication of the technological level of raw material processing achieved by prehistoric artists, related to powdering. Could these similarities between two distant territories reflect some sort of cultural relationship including not only sharing themes and images but also technologies of pigment processing? Submicrometric hematite can be available in nature [82,83] or result from anthropic manipulation (either grinding or firing) [49,50,84]). The characteristics of the materials identified in group 3 (low Fe % amount in clay matrix) are more likely related to a natural red ochre [2,11,41,59] which was probably only grinded. On the contrary, the high amount of pseudo-rounded submicrometric hematite identified in paint mixtures 1, 2 and the elongated shape detected in group 4 open the question of whether it could have resulted from firing any ferruginous raw materials. Thus, to determine whether Levantine artists were skilled grinders, or they even knew how to master the fire further geological surveys and analytical investigations following specific protocols are needed [50].

If we focus on group 1, characterized by the fine size of the hematite with dolomite, it is the first time that such composition has been found in Levantine art paints [11]. As already mentioned, it will be necessary to investigate in the future, through a systematic geological survey, whether any natural deposits rich in hematite and dolomite exist in the area surrounding the site, or whether the carbonate compound was intentionally added by the artists to create a particular paint recipe. The later practice was observed with the upper paleolithic red ochre from Lovas, Hungary, where anthropogenic processing of the natural pigment, called "core-shell" procession, has been suggested by Sajó et al. [85].

Regarding the colours, even though we have identified 4 paint recipes, they show two main colour hues at a macroscopic level. Motifs belonging to groups 1, 2 and 4 all show a purple color while those belonging to group 3 display a red hue, even though hematite is the main component in all of them. It is known that the color of hematite, thus its optical properties, depends on several factors: along with compositional aspects, as the presence of impurities and/or additional compounds (e.g., the presence of dolomite and clay matrix in groups 1 and 3 respectively), it also depends on hematite crystal morphology and size [86–88]. The hematite hue ranges from yellowish red for nanoparticles, to purple for micrometer-sized samples and even black for large opaque crystals. [87]. Such colors are due to the scattering and absorption of light in the blue–green part of the visible spectrum: when absorption exceeds scattering over the entire visible range, hence the Fe oxides appear darker [86,87]. Moreover, aggregation of individual small particles can affect the color as it does crystals size increase. This means that small hematite particles in aggregate behaves optically as large (purple) hematite particles [86,88,89]. All these evidences could explain why even though all 4 groups show submicrometric hematite, painting in group 3 look different (orange/red instead of purple) as the total amount of iron oxide in paintings belonging to this group is lower. Thus, with similar particle size, the higher the concentration of hematite, the darker the paintings appear.

### Interpreting calcium oxalates

Another interesting topic to discuss is the potential origin, nature and role of the calcium oxalates detected along the microstratigraphy of the samples (sandwiching or within the paint layers). The pictorial layer in all the investigated cross-sections is sandwiched between two compact strata of biogenic calcium-based material, whose molecular spectroscopic analyses showed the presence of calcium oxalates, always in the form of whewellite, and occasionally together with weddellite (S1 Tables 3 and 4 in S1 File). The presence of these compounds in rock art paintings is very common, and due to their stability and high insolubility in water, they can be considered an effective protective layer for the motifs [11,90].

Regarding their origin, in the open-air rock art context, where microorganisms like bacteria, lichens, fungi, etc. are currently living on the rock surface and are an integral part of it, these formations can be attributed to a biological origin (as confirmed also by the analyses of the external layers of samples AC10 and AC11). However, when the signal of oxalates is directly identified within the paint layers, as observed in the present study in which signals of whewellite have been also detected in the red pictorial layers of all the samples, it is important to consider the possibility that they can also result from the degradation of any organic binder used by the artists to prepare the paints, as the consequence of the oxidative processes of the organic material applied [46,91]. Oxalate salts have been frequently found within multiple paint layers in micro samples coming from easel paintings [91–93]. As a consequence, concerning the painting layers, both biological and chemical origins (from degradation of a binder) could contribute jointly to the calcium-oxalate formations within the red strata. Indeed, from this point of view, they can be considered as potential markers of the presence of an organic binder, now degraded, used for the creation of the art. The use of binders results reasonable and probable also if we consider the highly compactness and homogeneity of the pictorial layer in which the iron oxide is spread. Additionally, ethnoarchaeological research demonstrated the crucial role of binders in the long-term conservation of the rock art in the open air: paintings produced without them disappeared in a few years/decades from their exposure [94]. Moreover, the identification in most of the painting layers of calcium-based metal soaps can valerate the hypothesis that oxalates identified within the red paints layers can be considered as degradation products of the binder used by prehistoric artists. Likewise, oxalates, metal carboxylates are degradation compounds commonly found in paintings, that are the results of the interaction between binding organic media (such as siccative oils, proteinaceous binders, natural resins) and a metal cation, as in this case would be Ca from the rock [76]. Specifically, in both drying oils and proteinaceous binder like egg tempera, the metal soaps derive from the reaction between the free fatty acids in the medium (due to the hydrolysis of the triglycerides and from the egg yolk respectively) and the cations in the pigments [95,96]. Both binders can produce free fatty acids. In the case of natural resins like mastic, colophony, etc., the metal carboxylates arise by the reaction between the terpenic acids and the cations in the pigments [97,98]. Thus, if Levantine and Schematic prehistoric artists used any binder at el Carche, it would derive from one of these three classes of substances. Therefore, the formation of such degradation products (metal carboxylates) frequently affects paintings and represents a real issue from the conservation point of view [99]. Indeed, they can physically and mechanically damage the paint layer creating several kinds of problems in the stratigraphy, such as protrusions, delamination, cracks, etc. that can also lead to the detaching of the pictorial layer [75,99–101 to name few]. Besides factors like the specific paint composition and its stratigraphy, environmental conditions such as temperature, high level of relative humidity and introduction of water, highly contribute to their formation in the paint layer [76]. These are parameters to which rock paintings are constantly exposed and they cannot be controlled. The presence of such calcium-based metal soaps could be one of the reasons behind the presence of two different red layers in sample AC8 (despite belonging to a single motif 2), which show a similar composition. Another possible interpretation of the presence of two distinct pictorial layers belonging to a single motif, where no overlapping occurred, could be that the motif was repainted after a period in which an oxalate layer would have formed between the red strata. This observation would indicate a sequence of painting events starting with the depiction of the motif, that would eventually be repainted. However, based on our analyses, both the chemical composition, the texture, and appearance of the two red layers of sample AC8 are very similar if not identical. Therefore, it would seem more reasonable to think that something occurred to the paint layer that has induced a division, e.g., the breaking of the pictorial layer induced by the mechanical stress imparted by the formation of metal soaps, sulfates salts, etc. The presence of any cracks and fissures in the paint layers could had facilitated the inclusion of water from the surface that have triggered, as well, the formation of the interstratified Calcium-oxalate layers and sulfates strata. In this sample, calcium and/or strontium sulfate strata are highly spread also within the paint layer, and they often occur parallelly among the two divided red levels (Fig 8H, magenta), forming physical patterns and fissures like channels [16].

More complex is the evaluation of the interstratified strata observed in samples AC4 and AC5.2. Considering that we have been unable to establish the order of overlap between motifs 10 and 11 (or even to clarify the presence of an additional third motif or repaint), we still cannot discard any of the potential formation patterns explained above to interpret their presence. Thus, also in this case, further studies of the samples are required.

Beyond these specific samples, the microstratigraphic analyses showed that paints were applied on existing biogenic patinas developed on the rock surface and that, once painted, new patinas of biological origin continued to grow over the painted surfaces. This is why the paint layers appear between levels of Ca-rich materials (namely, oxalate, carbonate, sulphate and some phosphate). Today, paints coated by these patinas are protected against decay due to atmospheric agents like run-off water, moisture, air borne, etc. It is very likely that these very ancient paints have survived to the present also thanks to these natural protection [90,102].

## Conclusions

Our recent multi-technical and multi-step analysis of the prehistoric paintings of el Carche rock shelter have yielded interesting new data on the variety of sources, paint compositions, transformation technologies, sequence of events (painting and non-painting phases, as well as potential repainting events) and on some deterioration and conservation issues related to Levantine and Schematic rock art. Thanks to these analyses, we have been able to reflect on and draw conclusions of interest both for studies of Levantine and Schematic art in the Iberian Peninsula and for prehistoric rock art more globally, and especially for open-air rock art.

The analyses show that Levantine art was produced using four different pigment compositions, while Schematic art shows two types. Surprisingly, as already observed by Mas et al. [25], at some stage Levantine artists used similar colour hues and pigment compositions to Schematic artists. Such similarities could arise from the use of the same source of supply over time or the use of similar processing technologies and raw materials. This second option could involve some sort of cultural connections both among several Levantine phases (as pigments in group 3 belong to Levantine motifs of different substyles and phases, that even overlap) and between Levantine and Schematic artists. Hopefully, future geological surveys aimed at identifying potential sources of supply will help us answering this question. Nevertheless, this paper demonstrates that both Levantine and Schematic artists used a variety of raw material sources or maybe paint recipes, thus challenging previous assumptions suggesting that the chemical composition of Levantine and Schematic paintings was similar [25,103].

On the other hand, variability in paint composition among Levantine paintings suggests that Levantine artists used also a variety of sources and/or processing techniques. This variety of pigment sources or recipes, together with the stylistic variability and overlaps between Levantine motifs, as well as between those and Schematic art suggests that this site remained culturally significant over a certain period of time.

The results have also opened new questions that were unthinkable before this analytical approach. The open questions include whether the presence of Carboxylates and oxalate signals identified within the pictorial layers could be related to the degradation of binders, or whether the reduced particle size of the paints of some paint groups is natural or it is an indicator that Levantine artists were skilled grinders, or they even master the fire. These questions are not yet resolved and will be further addressed in our future research.

The analyses performed have also shown a new step in the creation process that went unnoticed in our previous visual analyses of the motifs and scenes. The composition of the paints of motifs 2 (deer) and 4 (hunter), interpreted as part of a hunting scene, does not match. This difference in pigment composition challenges our previous interpretation as simultaneous motifs [28]. Whether the two layers identified in sample from deer 2 reflect a possible repaint, that may or may not have been concurrent to the addition of the archer, or a conservation problem, will have to be investigated in the future with further in situ and in lab analysis.

The results of the project also draw attention to the value of stratigraphic analyses, which only rarely have been performed on Levantine rock art [11]. In this research, our stratigraphic analyses have been useful not only to establish the sequence of painting events (most successfully in the superimpositions among motifs 5, 6, 7 and 9 than in that of motifs 10 and 11), but also to reflect on the time elapsed between overlapped motifs. Regarding these latter, three behaviors have been observed:

- no crust formation between two different and adjacent paint layers (AC1 and AC3), suggesting perhaps a short or non-existent time lapse between them;
- in some apparent areas of overlap only a single layer of paint has been preserved (AC2 and AC5.1), indicating that perhaps when a new motif was added, the paint of the lower motif had already disappeared in that particular area.
- Ca-rich interstratified strata between different paint layers (AC4 and AC5.2), suggesting, among other hypotheses, a certain time span between interventions.

Unfortunately, not knowing the rate of patina formation complicates drawing stronger conclusions. The unexpected behavior of our samples (3 layers were expected in AC1 and two layers in AC2, AC4 and AC5, but they were not documented) illustrate well the challenges of sampling and interpreting areas of overlap.

Surprisingly, in a sample collected from a single motif (AC8) an interstratified oxalate layer was identified within the paint layer. In this case, considering that it is just one motif, and the composition of the paint is exactly the same in both layers, we conclude that more than resulting from a certain time span between interventions (e.g., prehistoric repainting), the oxalate layer is more likely the product of a degradation process. Further studies will be necessary to understand the main factors triggering the separation. Whether the interstratified crust formations observed in samples AC4 and AC5.2 result from alterations of the painted layer or a time span between painting events will be further explored in the future.

Moreover, the micro-stratigraphic analysis of the samples confirmed once more the protective role of the external patinas covering the paints [77]. This brings us back to the concern and the debates about cleaning conservation interventions, given that it has been common practice in these territories to reduce their thickness or even to remove them to improve visualization. It therefore becomes urgent to develop appropriate protocols to protect the paintings and their scientific values if this sort of interventions is going to continue in the future.

Finally, beyond the interest of the analytical results offered in this study, another important contribution of this paper is to demonstrate the relevance of developing well-structured analytical protocols to maximize results while minimizing impact on this unique and irreplaceable heritage. Based on our experience, the ideal scenario would be to combine complementary analytical techniques at different scales, linking elemental and molecular results with morphological evaluations. So far, our multi-step, multi-scale and multi-analytical approach has proven effective, with a first stage of on-site non-invasive EDXRF analysis (useful to first group the paints according to their constitutive elements), which has been valuable in guiding a second phase of microsample extraction and analyses, using mainly non-destructive spectroscopic techniques [2]. Of the methods chosen, the use of FTIR showed more technical problems related to resin penetration.

In our opinion, such an analytical approach could be considered the ideal protocol to be used globally as a guideline, as it meets the challenge of finding a balance between advancing scientific knowledge and the need to preserve this heritage for future generations.

## Supporting information

S1 File Contains all the supporting figures and tables. (PDF)
