# Supplementary material for: Characterizing paint technologies and recipes in Levantine and Schematic rock art: El Carche site as a case study (Jalance, Spain)
Source: PLoS One. 2022 Aug 15;17(8):e0271276. doi: 10.1371/journal.pone.0271276 (PMC9377580; doi:10.1371/journal.pone.0271276)
Supplement: S1 File — (PDF) [file pone.0271276.s001.pdf]

S1 File – Supporting Information – Contains all the supporting figures and tables

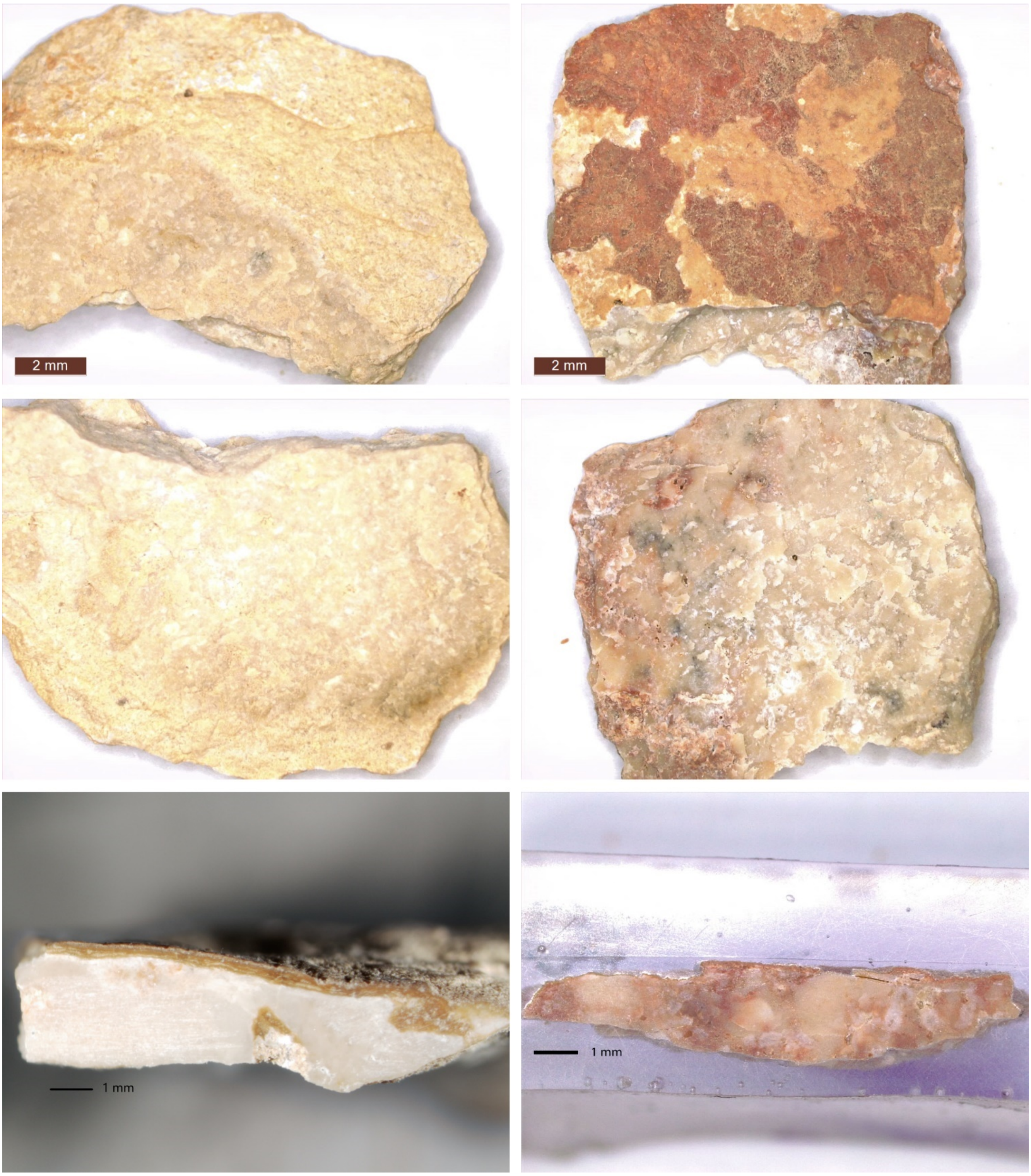

Fig 1. From top to bottom: images of top, back sides and cross-section of substrate samples AC10 (left column) and AC11 (right column).

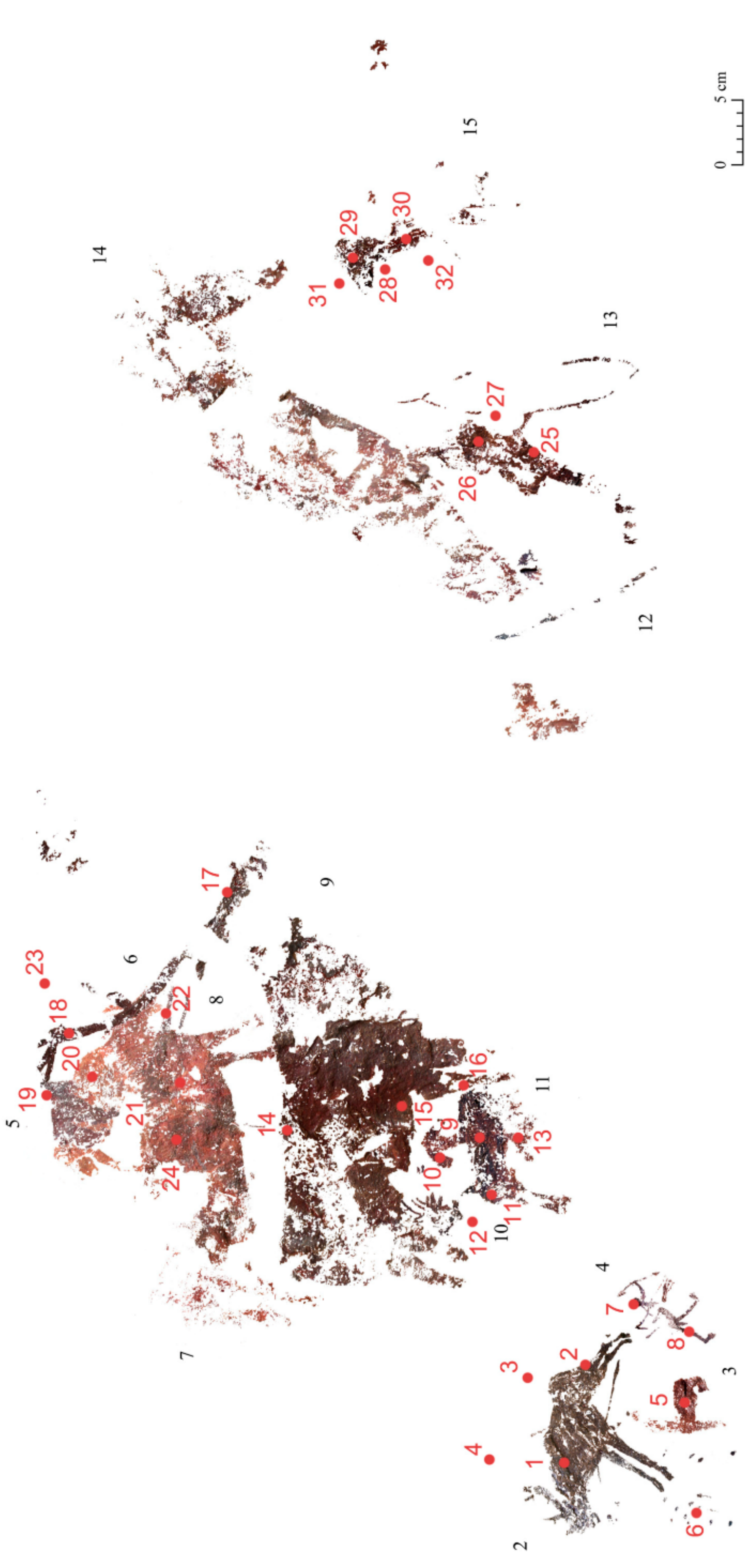

Fig 2. Digital tracing of the Levantine and Schematic paintings of el Carche rock art site. In red, the points where the in situ EDXRF analyses were performed. Black numbers refer to motif numbers.

Table 1. Normalized net areas corresponding to the most significant elements detected by EDXRF point analyses of red motifs and the substrate.

Points and Location*	S (Kα)	±	K (Kα)	±	Ca (Kα)	±	Ti (Kα)	±	Mn (Kα)	±	Fe (Kα)	±	Sr (Kα)	±
**1 – motif 2**	0.0049	0.0002	0.0084	0.0003	0.330	0.002	0.0015	0.0001	0.0016	0.0001	0.1822	0.0015	0.0871	0.0010
**2 – motif 2**	0.0069	0.0003	0.0067	0.0003	0.295	0.002	0.0011	0.0001	0.0016	0.0001	0.2145	0.0018	0.1088	0.0012
**3 – substrate**	0.0050	0.0002	0.0098	0.0004	0.448	0.003	0.0007	0.0001	0.0001	0.00004	0.0339	0.0007	0.0836	0.0011
**4 – substrate -fresh**	0.0190	0.0005	0.0058	0.0003	0.467	0.003	0.0006	0.0001	0.0005	0.0001	0.0198	0.0005	0.0783	0.0010
**5 – motif 3**	0.0048	0.0003	0.0079	0.0003	0.340	0.003	0.0021	0.0002	0.0011	0.0001	0.0690	0.0010	0.1606	0.0016
**6 – motif 2 vomit**	0.0066	0.0003	0.0071	0.0003	0.387	0.003	0.0015	0.0001	0.0009	0.0001	0.0773	0.0010	0.1156	0.0012
**7 – motif 4**	0.0049	0.0002	0.0074	0.0003	0.313	0.002	0.0015	0.0001	0.0003	0.00005	0.0893	0.0010	0.1957	0.0016
**8 – motif 4**	0.0035	0.0002	0.0075	0.0003	0.246	0.002	0.0014	0.0001	0.0008	0.0001	0.0576	0.0007	0.3528	0.0020
**9 – motifs 10-11**	0.0050	0.0002	0.0082	0.0003	0.307	0.002	0.0013	0.0001	0.0015	0.0001	0.2036	0.0015	0.1196	0.0011
**10 – motif 11**	0.0064	0.0003	0.0080	0.0003	0.386	0.003	0.0003	0.0001	0.0006	0.0001	0.1158	0.0012	0.0790	0.0010
**11 – motif 10**	0.0083	0.0003	0.0064	0.0002	0.333	0.002	0.0014	0.0001	0.0009	0.0001	0.1016	0.0010	0.1797	0.0014
**12 – substrate**	0.0042	0.0002	0.0100	0.0003	0.468	0.003	0.0014	0.0001	0.0005	0.0001	0.0308	0.0006	0.0669	0.0009
**13 – motif 11**	0.0092	0.0003	0.0071	0.0003	0.351	0.002	0.0008	0.0001	0.0005	0.0001	0.0460	0.0007	0.2044	0.0016
**14 – motif 9**	0.0046	0.0002	0.0062	0.0003	0.295	0.002	0.0010	0.0001	0.0020	0.0001	0.1946	0.0016	0.1243	0.0012
**15 – motif 9**	0.0027	0.0002	0.0087	0.0003	0.366	0.002	0.0009	0.0001	0.0016	0.0001	0.1765	0.0016	0.0565	0.0008
**16 – motif 9**	0.0052	0.0002	0.0098	0.0003	0.398	0.002	0.0009	0.0001	0.0004	0.0001	0.1083	0.0011	0.0804	0.0009
**17 – motif 9**	0.0177	0.0004	0.0052	0.0002	0.409	0.002	0.0002	0.00004	0.0011	0.0001	0.1196	0.0011	0.0753	0.0009
**18 – motif 9**	0.0079	0.0003	0.0065	0.0003	0.364	0.002	0.0004	0.0001	0.0019	0.0001	0.1489	0.0013	0.0911	0.0010
**19 – motif 5**	0.0195	0.0005	0.0063	0.0003	0.377	0.002	0.0004	0.0001	0.0008	0.0001	0.1030	0.0011	0.0869	0.0010
**20 – motif 6**	0.0135	0.0004	0.0061	0.0003	0.400	0.003	0.0004	0.0001	0.0001	0.00003	0.0376	0.0007	0.1286	0.0013
**21 – motif 7**	0.0083	0.0003	0.0072	0.0003	0.424	0.003	0.0009	0.0001	0.0006	0.0001	0.0513	0.0008	0.1038	0.0011
**22 – motif 8**	0.0274	0.0006	0.0062	0.0003	0.416	0.003	0.0006	0.0001	0.0008	0.0001	0.0581	0.0009	0.0652	0.0009
**23 – substrate**	0.0067	0.0003	0.0062	0.0003	0.431	0.003	0.0008	0.0001	0.0006	0.0001	0.0178	0.0005	0.1302	0.0013
**24 – motif 7**	0.0129	0.0004	0.0069	0.0003	0.424	0.003	0.0008	0.0001	0.0004	0.0001	0.0447	0.0007	0.0984	0.0011
**25 – motif 13**	0.0303	0.0006	0.0074	0.0003	0.385	0.002	0.0016	0.0001	0.0009	0.0001	0.1022	0.0011	0.0623	0.0008
**26 – motif 13**	0.0207	0.0004	0.0077	0.0003	0.330	0.002	0.0017	0.0001	0.0011	0.0001	0.1129	0.0011	0.1412	0.0012
**27 – substrate-fresh**	0.0239	0.0005	0.0073	0.0003	0.452	0.003	0.0013	0.0001	0.0008	0.0001	0.0289	0.0006	0.0685	0.0009
**28 – substrate-orange**	0.0176	0.0004	0.0044	0.0002	0.210	0.001	0.0005	0.0001	0.0002	0.00004	0.0240	0.0004	0.4359	0.0020
**29 – motif 15**	0.0563	0.0009	0.0030	0.0002	0.304	0.002	0.0001	0.00003	0.0005	0.0001	0.0598	0.0009	0.1543	0.0015
**30 – motif 15**	0.0236	0.0005	0.0052	0.0002	0.300	0.002	0.0007	0.0001	0.0012	0.0001	0.1396	0.0013	0.1628	0.0015
**31 – substrate-orange**	0.0255	0.0005	0.0056	0.0002	0.391	0.002	0.0009	0.0001	0.0007	0.0001	0.0286	0.0006	0.1527	0.0014
**32 – substrate-white**	0.0561	0.0008	0.0028	0.0002	0.356	0.002	0.0001	0.00004	0.0011	0.0001	0.0097	0.0003	0.1612	0.0015
**33 – substrate**	0.0204	0.0005	0.0064	0.0003	0.388	0.002	0.0008	0.0001	0.0005	0.0001	0.0405	0.0007	0.0454	0.0007

*The location of each point within the panel is shown in S1 Fig.

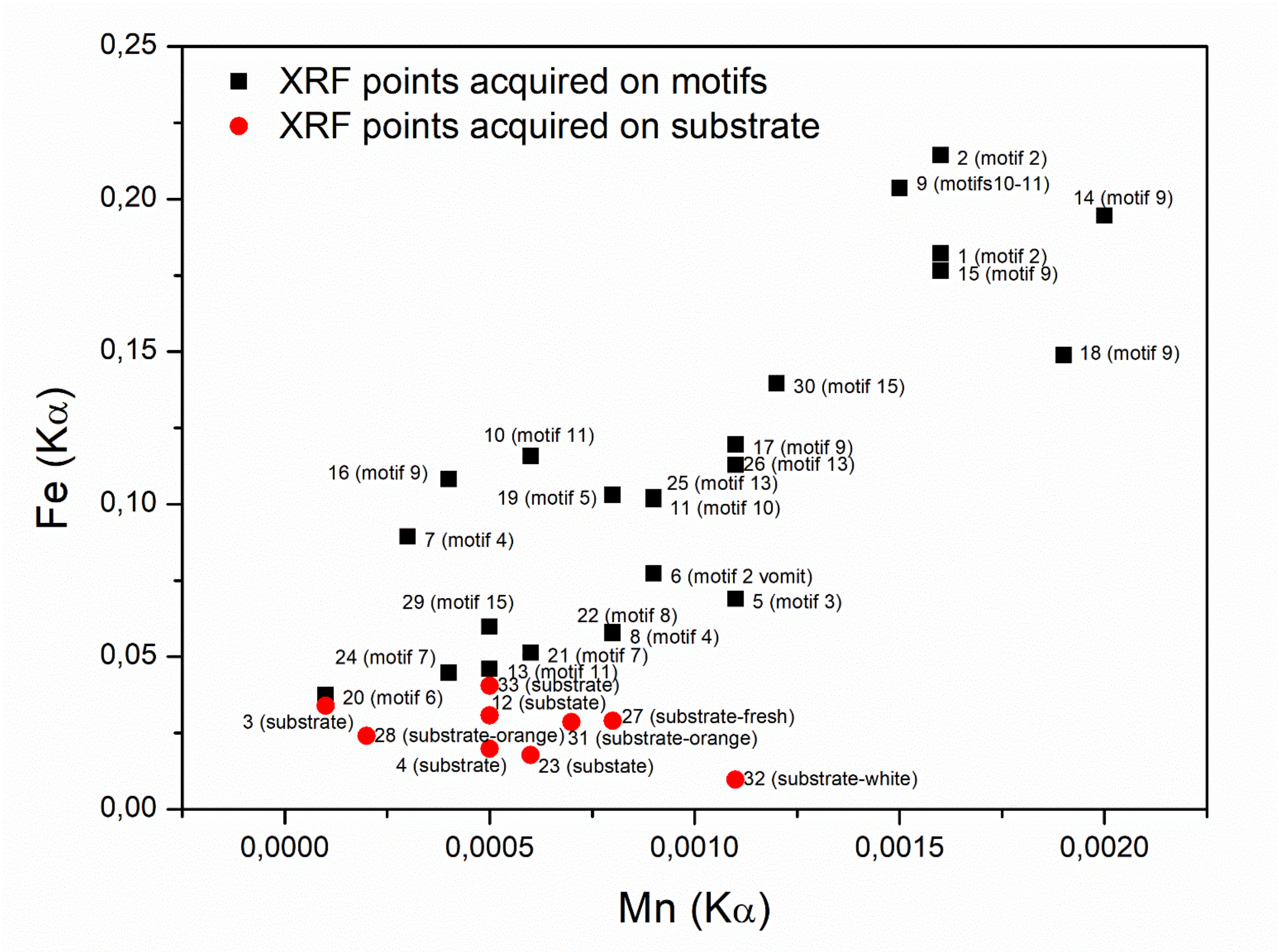

Fig 3. Normalized net areas of the Fe-Kα fluorescence lines *versus* the normalized net areas of the Mn-Kα fluorescence lines from the in-situ EDXRF point analyses performed both on the motifs (black squares) and on the substrate (red circles) at el Carche site.

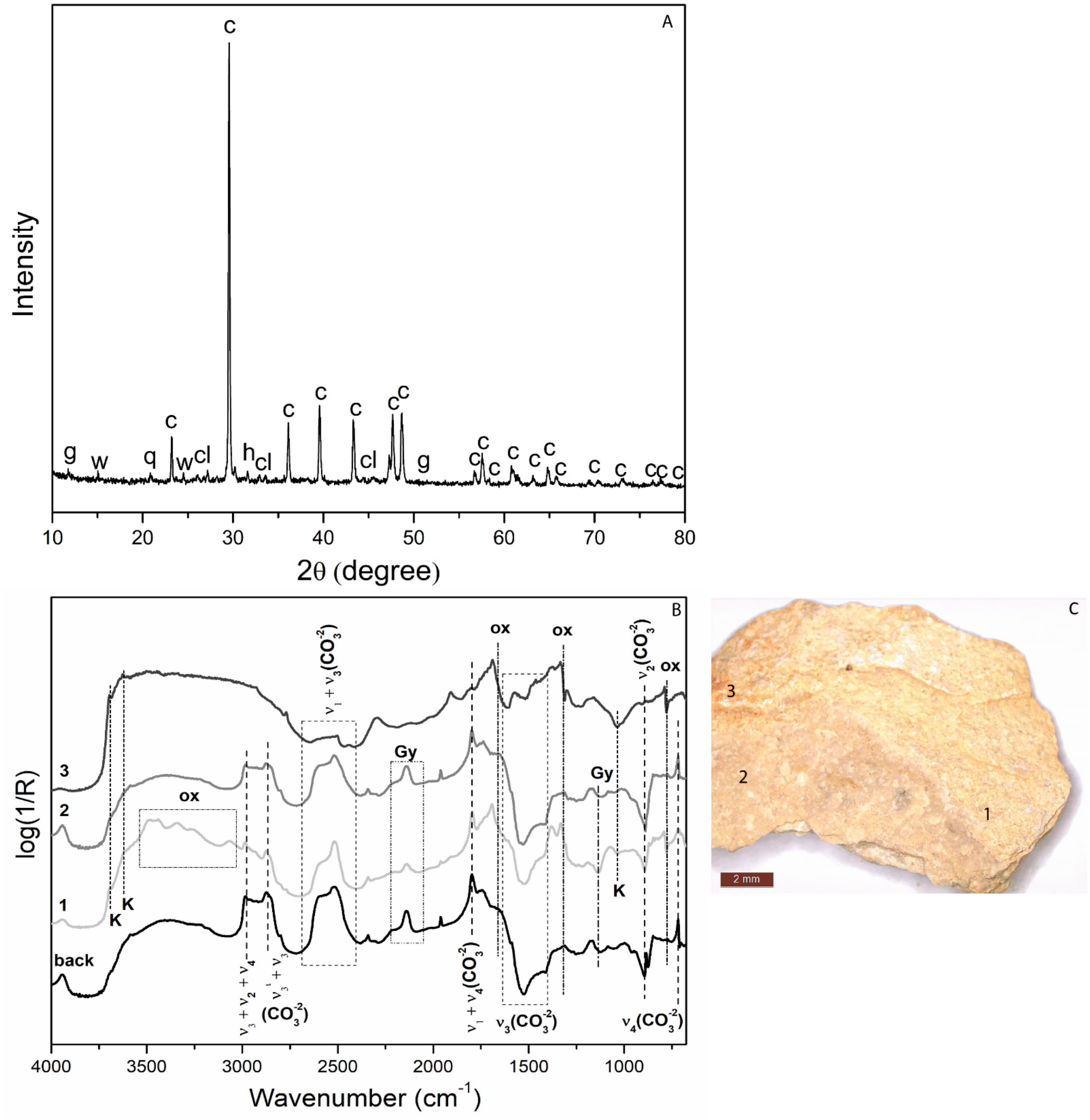

Fig 4. A) Diffractogram relative to sample AC10. Legend: c= calcite; q= quartz; g= gypsum; h= hydroxyapatite; cl= celestite; w= whewellite. B) FTIR reflectance spectra acquired on the surface of sample AC10. The points analyzed are shown in image C (1-3). A spectrum from the back side of the sample has also been collected. Legend: ox= Ca-oxalate (whewellite-like); gy= gypsum; k= kaolin. In image B, the strong absorption in the region 1450–1420 cm^{-1} related to the antisymmetric stretching (ν_3) of carbonate group (CO_3^{-2}) of calcite, is inverted by the reststrahlen effect, while the bands relative to the out-of-plane bending (ν_2) and to in plane-bending (ν_4) at 890 cm^{-1} and 715 cm^{-1} have a derivative shape. These spectral distortions are typical when working in reflectance configuration. Combination bands ($\nu_x + \nu_y$) of carbonate group (CO_3^{-2}) are also reported in the graph [1, 2]. The FTIR analyses showed also signals of calcium-oxalates, whewellite like. They present bands (sometimes derivative-like) at ca. 1650, 1320, and 775 cm^{-1} that are assigned to the CO antisymmetric and symmetric stretching mode of the oxalate anion, ν_a(CO) and ν_s(CO), and the bending mode δ(OCO) of whewellite, respectively [3]. The asymmetric stretching band (ν_3) of S-O characteristic of gypsum is also present at ca. 1165 cm^{-1} as well as its the combination bands of $\nu_1 + \nu_3$ at ca 2100-2300 cm^{-1} [1, 4]. Finally, Si–O stretching mode at 1100–1010 cm^{-1} and OH stretching at 3600 cm−1 have been assigned to kaolin [1].

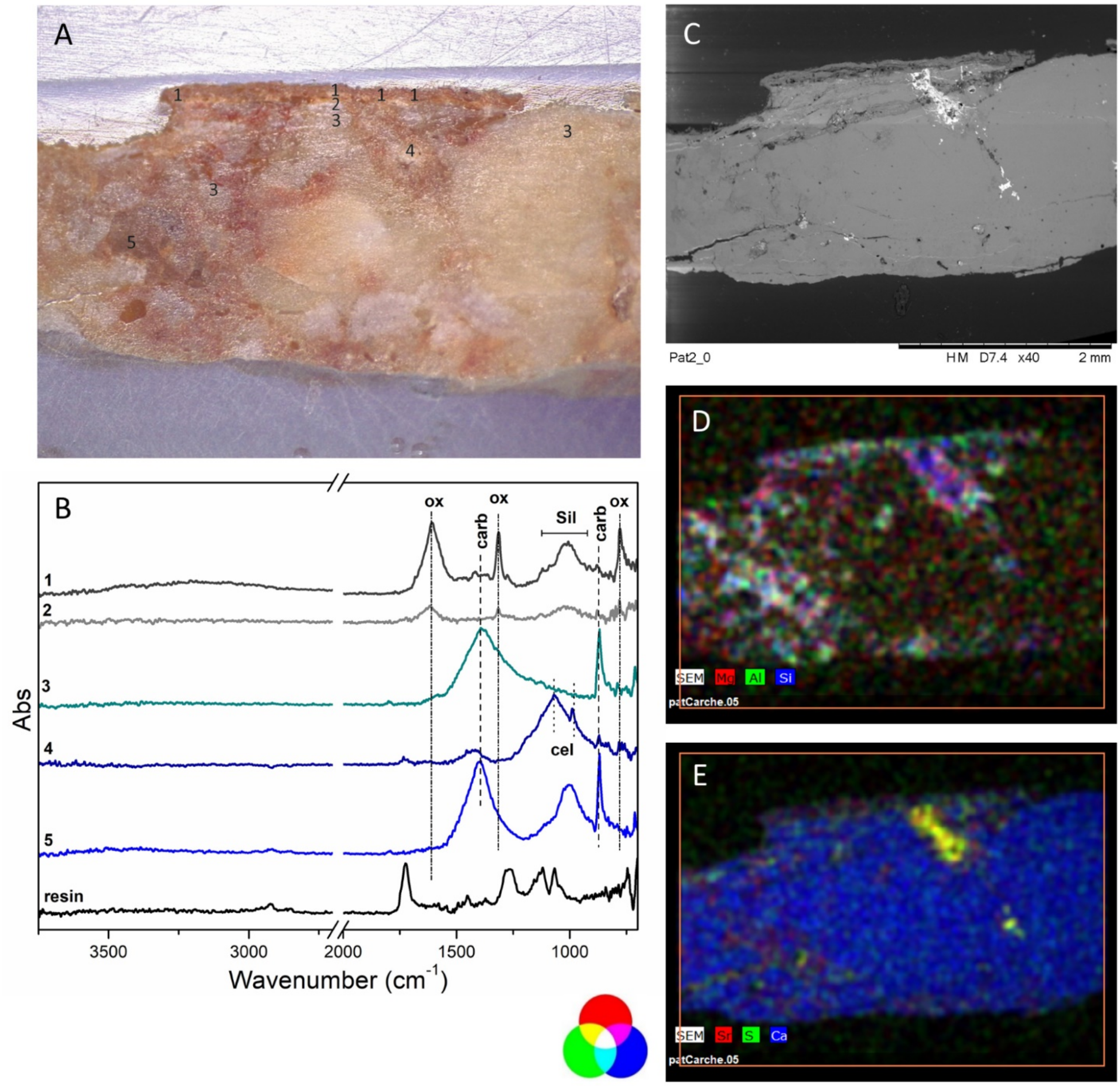

Fig 5. A) OM image of a selected area of sample AC11. The numbers refer to the ATR spectra acquired in the samples and shown in B). C) SEM backscattered image and their RGB composite images of the elemental distribution of D) $Mg_{K\alpha}/Al_{K\alpha}/Si_{K\alpha}$ and E) $Sr_{L\alpha}/S_{K\alpha}/Ca_{K\alpha}$ of the selected area of cross-sections AC11 shown in A. Legend: ox= Ca-oxalate (whewellite-like); carb= calcium carbonate (calcite-like); sil= silicate stretching signals; cel= Sr-sulfate, (celestite, $SrSO_4$). Five different groups of spectra have been identified in the sample. Their positions are shown in image A. The spectra named 1 and 2, have been collected close to the external surface and are characterized by Ca-oxalates (bands at ca. 1605, 1320, and 779 cm^{-1} that are assigned to the CO antisymmetric and symmetric stretching mode of the oxalate anion, ν_a(CO) and ν_s(CO), and the bending mode δ(OCO) of whewellite, respectively) and silicate signals (Si–O stretching at ca. 1100–1010 cm^{-1}) [1-3]. Those named 3 are mainly characterized by signals of calcite (bands at ca. 1395 cm^{-1} and 868 cm^{-1} relative to ν_3 antisymmetric stretching of (CO_3^{-2}) and ν_2 out-of-plane bending respectively [1,2]). Spectrum 4 is characteristic of celestite, $SrSO_4$, and it has been collected in the whitish areas rich in Sr (S-O stretching mode ν_3 at 1065 cm^{-1} [5]). Finally, group 5 spectra are characterized by both calcite and silicate signals and they are characteristics of the more brownish part of the samples. The FTIR spectra are in good agreements with the SEM-EDX mapping.

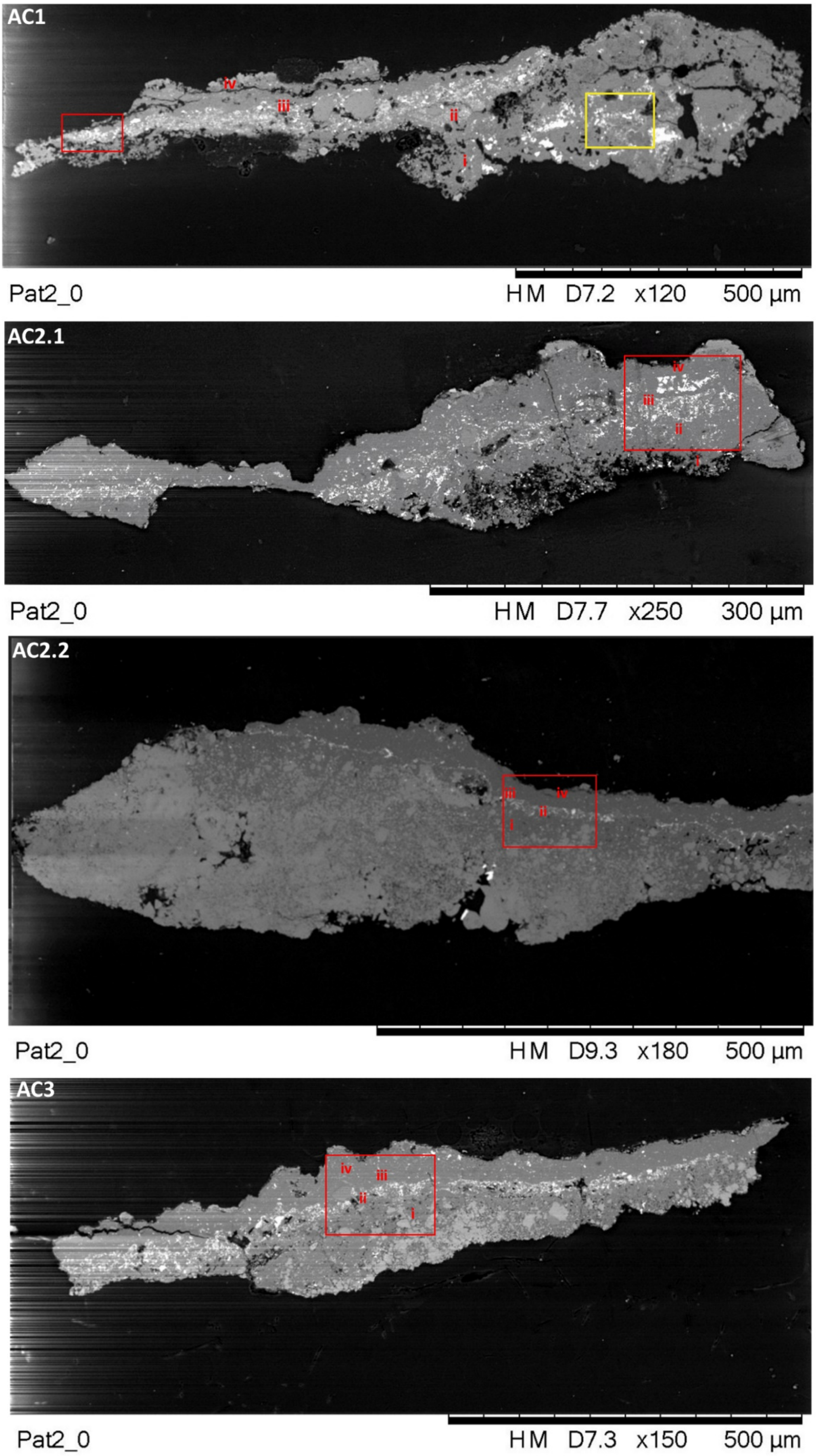

Fig 6. SEM images of cross-sections AC1 – AC3 in back scattered electron mode. The names of the samples are reported in the upper left part of each image. In the SEM images the four representative layers characterizing the microstratigraphy, namely i) external crust, ii) red pictorial layer, iii) intermediate coating covering the iv) substrate, are highlighted. Red and yellow squares show the areas where microanalyses have been performed.

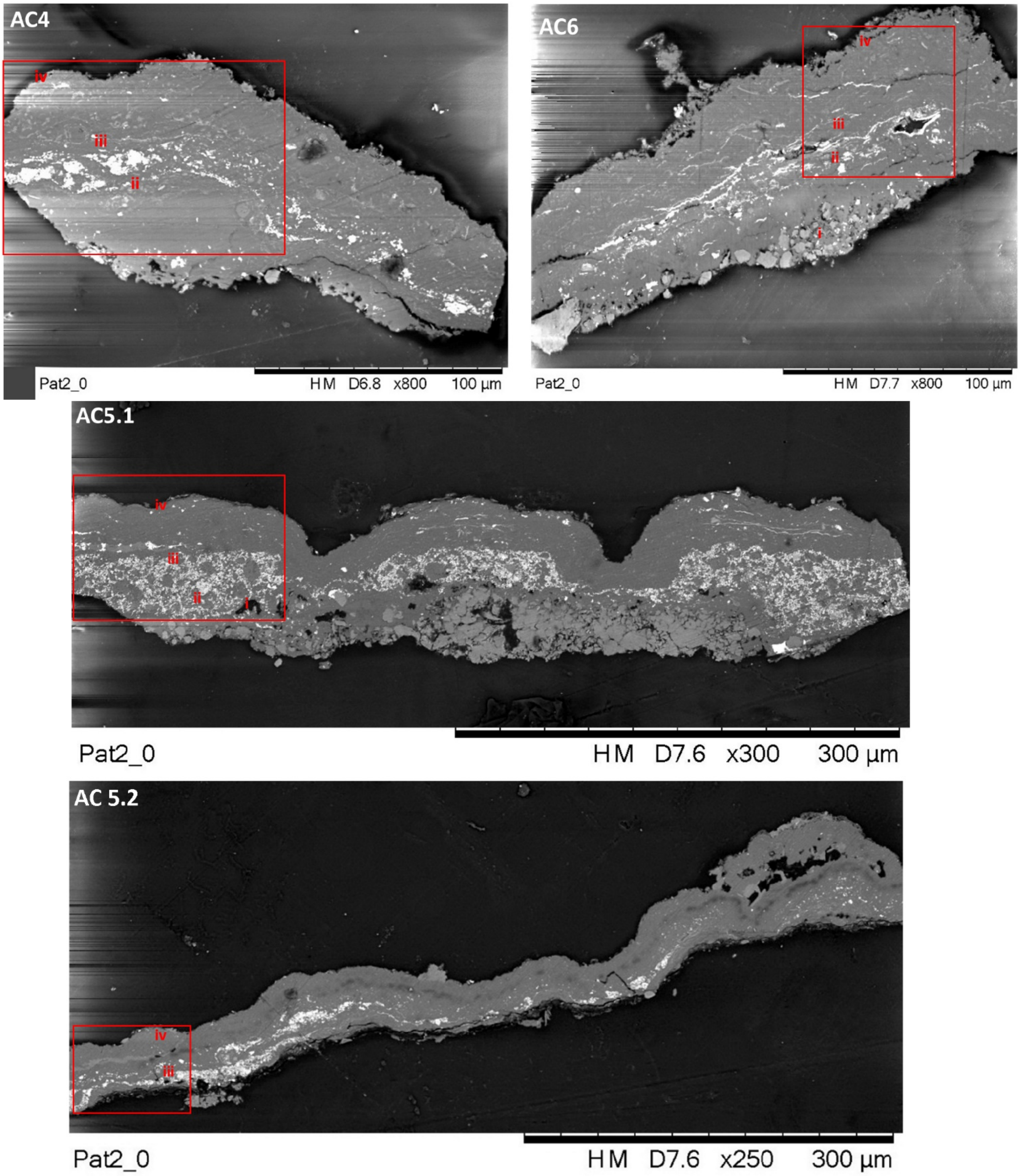

Fig 7. SEM images of cross-sections AC4 – AC6 in back scattered electron mode. The names of the samples are reported in the upper left part of each image. In the SEM images the four representative layers characterizing the microstratigraphy, namely i) external crust, ii) red pictorial layer, iii) intermediate coating covering the iv) substrate, are highlighted. In sample AC4 only strata ii, iii and iv are present, while in sample AC5.2 only layers iii and iv are visible. Red squares show the areas where microanalyses have been performed.

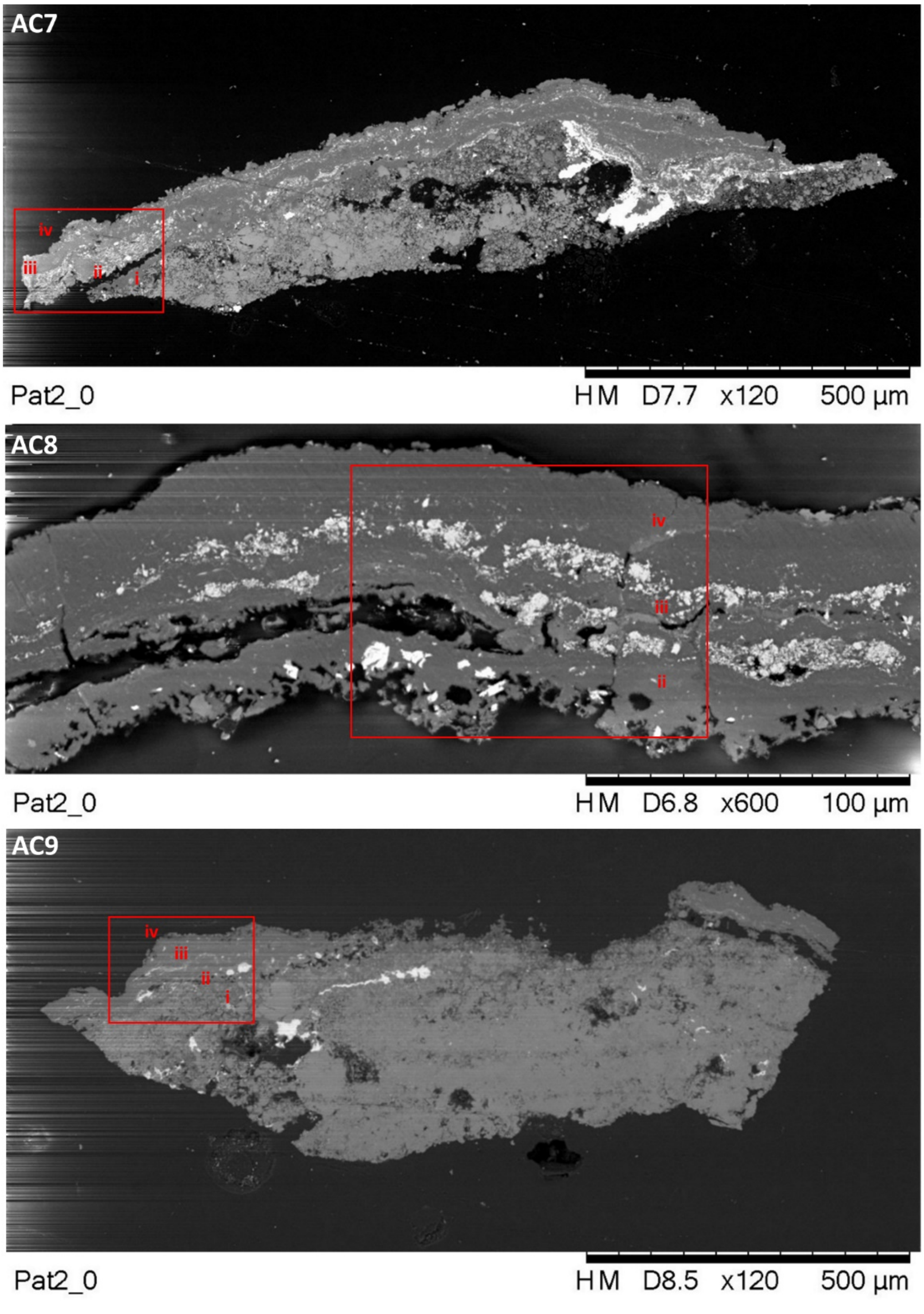

Fig 8. SEM images of cross-sections AC7 – AC9 in back scattered electron mode. The names of the samples are reported in the upper left part of each image. In the SEM images the four representative layers characterizing the microstratigraphy, namely i) external crust, ii) red pictorial layer, iii) intermediate coating covering the iv) substrate, are highlighted. In sample AC8, only strata ii, iii and iv are present. Red squares show the areas where microanalyses have been performed.

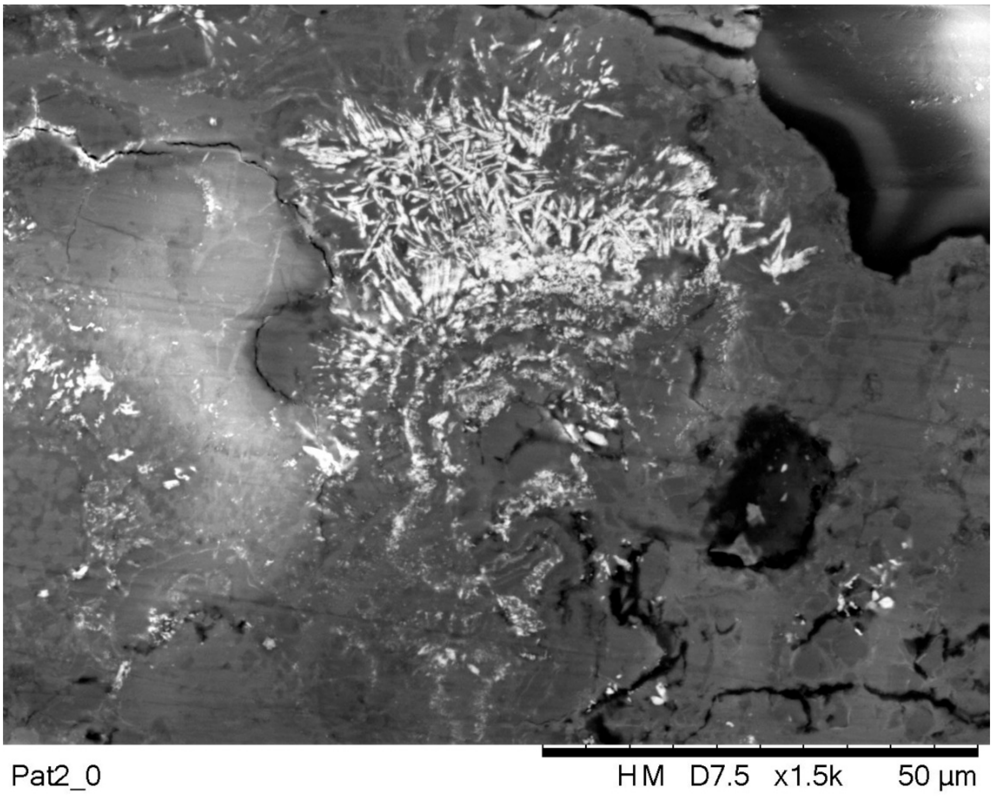

Fig 9. SEM image in back scattered electron mode of a selected portion of cross-section AC1 where needle-like crystal of celestite ($SrSO_4$) are displayed. The exact position of the analyzed area is highlighted in yellow in the top image of figure S5.

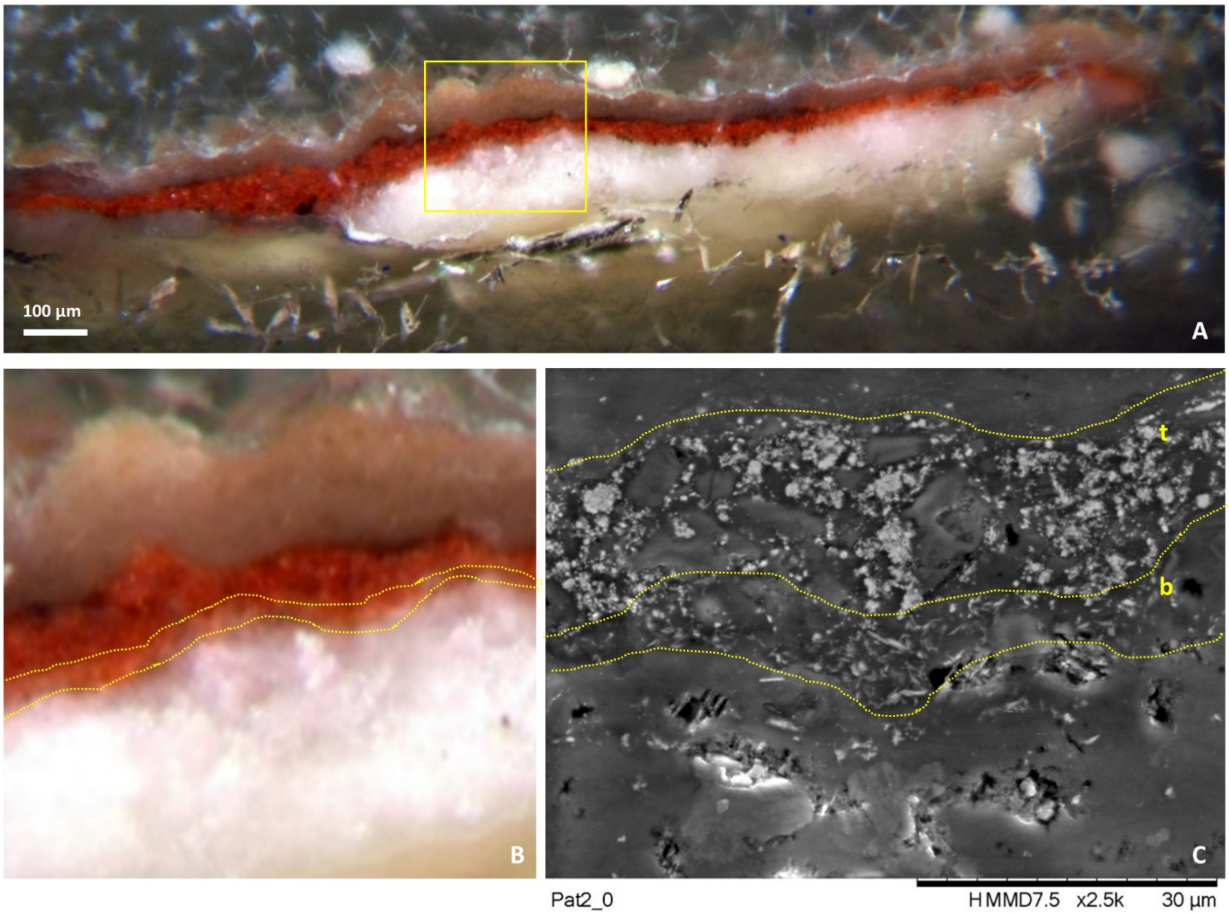

Fig 10. A) Optical microscope image, B) its zoomed area with C) its corresponding SEM backscattered image of cross-section AC3. In image C, the highlighted area shows the presence of two different pictorial layers, named t (top) and b (bottom).

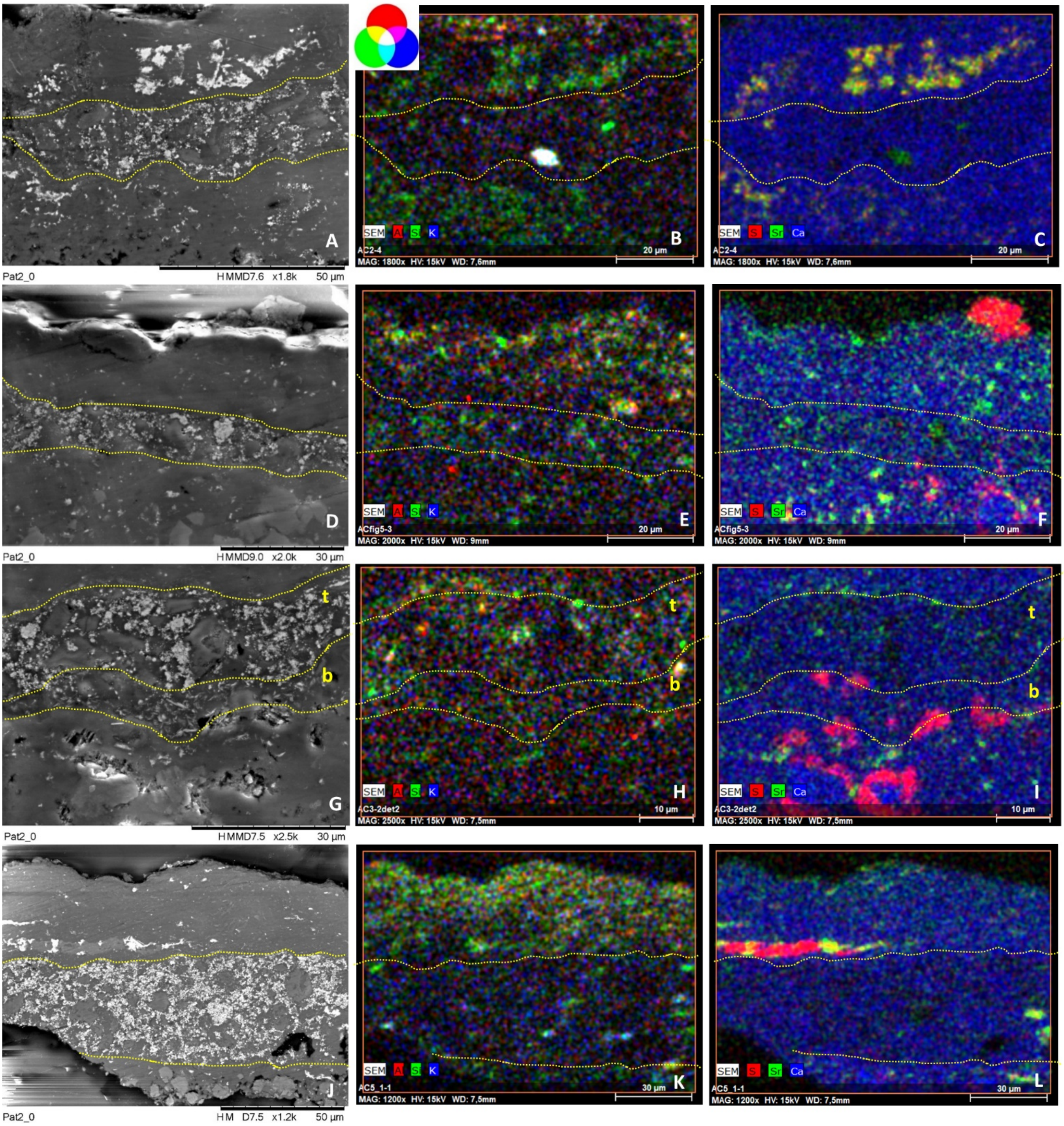

Fig 11. SEM backscattered images (left column) and their RGB composite images of the elemental distribution of $Al_{K\alpha}/Si_{K\alpha}/K_{K\alpha}$ (central column) and $S_{K\alpha}/Sr_{L\alpha}/Ca_{K\alpha}$ (right column) of a selected area of cross-sections AC2.1 (images A, B, C), AC2.2 (images D, E, F), AC3top (images G, H, I), AC5.1 (images J, K, L) constitutive of group 1. See S6 – S8 Figs to visualize the location of the analyzed areas (framed by red squares).

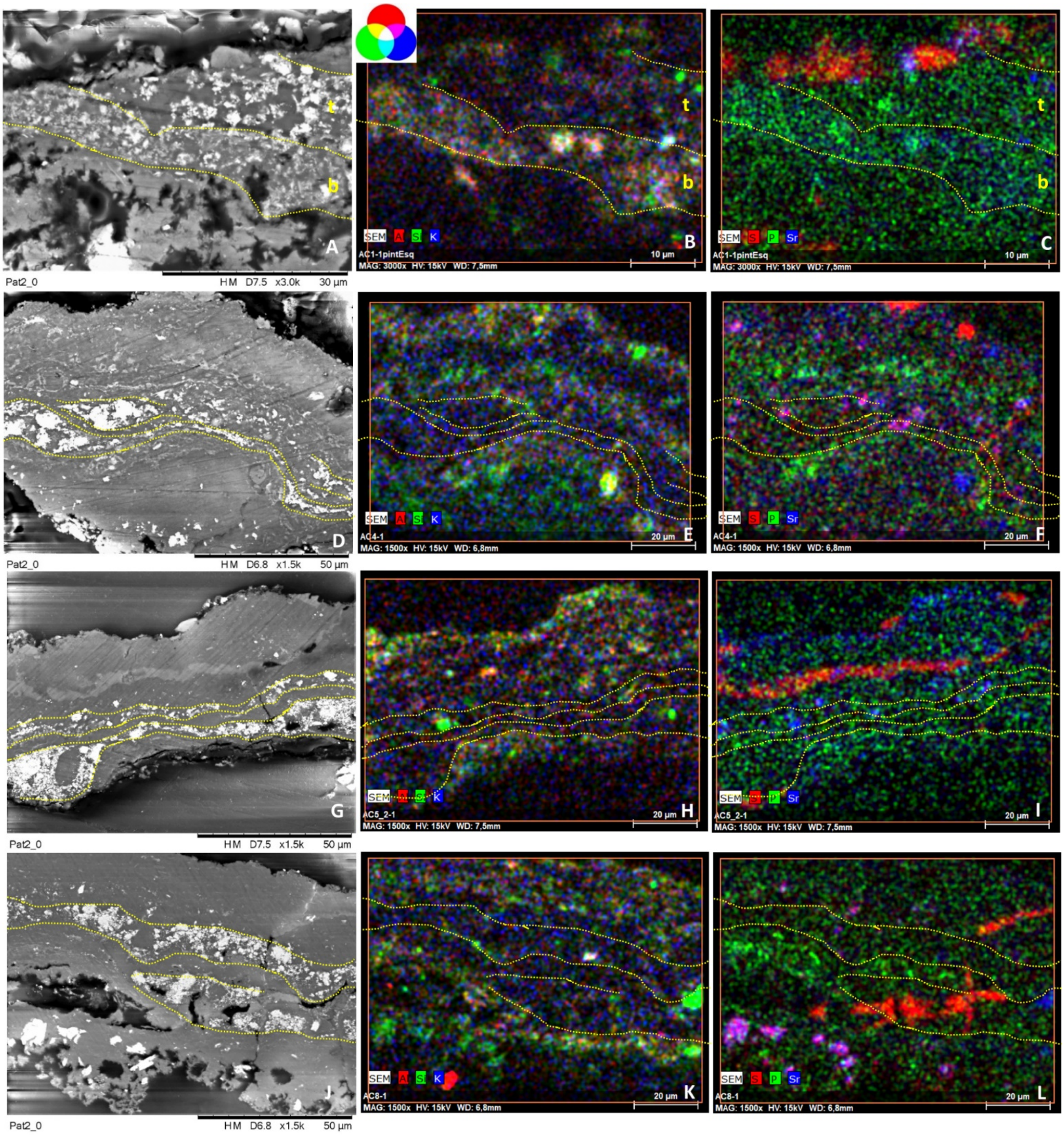

S12 Fig. SEM backscattered images (left column) and their RGB composite images of the elemental distribution of $Al_{K\alpha}/Si_{K\alpha}/K_{K\alpha}$ (central column) and $S_{K\alpha}/P_{K\alpha}/Sr_{L\alpha}$ (right column) of a selected area of cross-sections $AC1_{top}$ (images A, B, C), AC4 (images C, D, E), AC5.2 (images G, H, I), AC8 (images J, K, L) constitutive of group 2. See S6 – S8 Figs to visualize the location of the analyzed areas (framed by red squares).

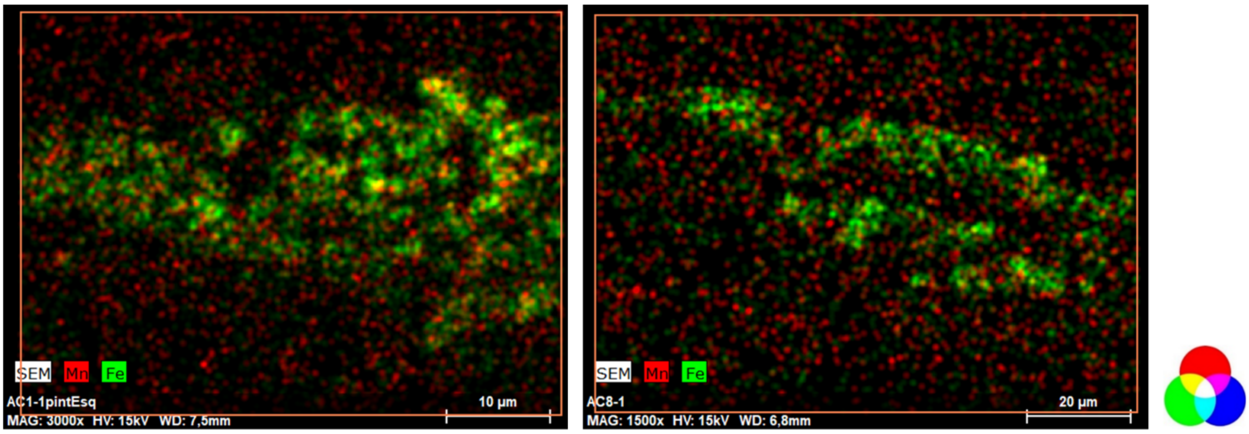

Fig 13. RGB composite images of the elemental distribution of $Mn_{K\alpha}/Fe_{K\alpha}$ of a selected area of the cross-sections AC1 (left) and AC8 (right). See S5 and S6 Figs to visualize the location of the analyzed areas (framed by red squares).

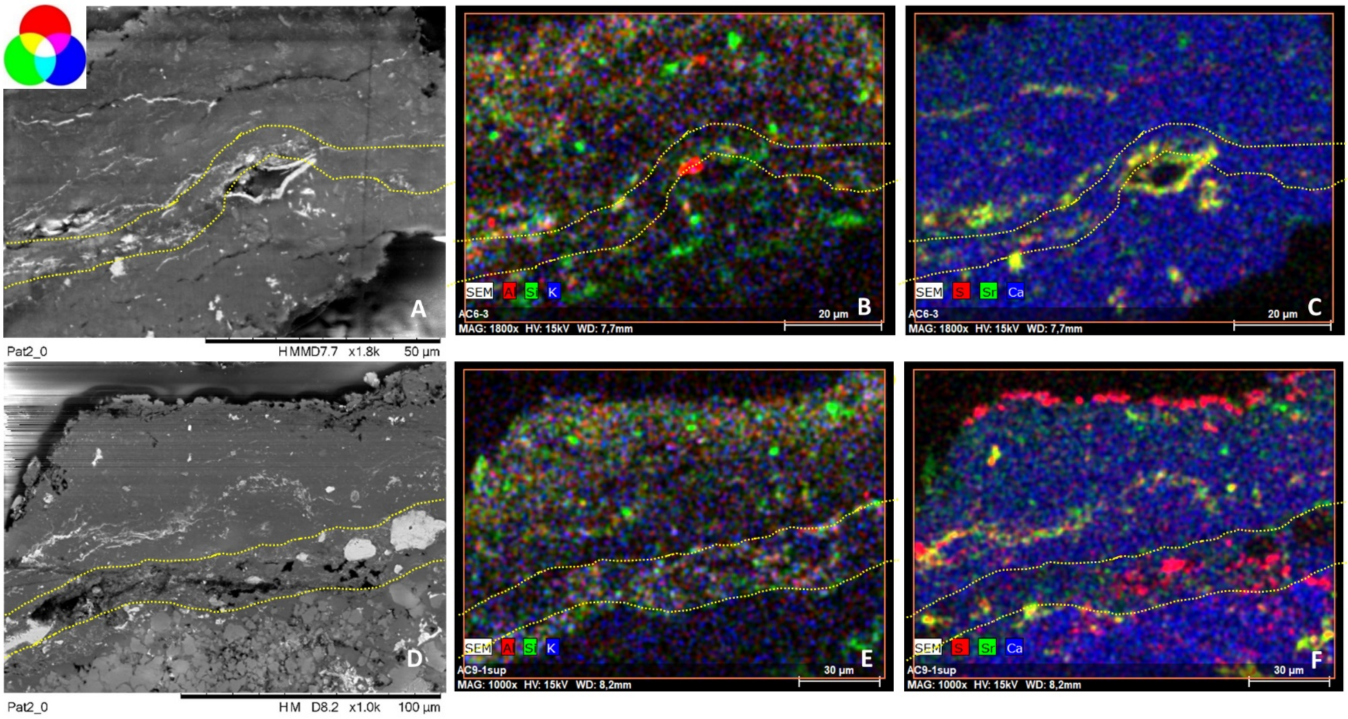

Fig 14. SEM backscattered images (left column) and their RGB composite images of the elemental distribution of $Al_{K\alpha}/Si_{K\alpha}/K_{K\alpha}$ (central column) and $S_{K\alpha}/Sr_{L\alpha}/Ca_{K\alpha}$ (right column) of a selected area of the cross-sections AC6 (images A, B, C) and AC9 (images D, E, F) constitutive of group 3. The mapping relative to the bottom layer of cross-sections AC1 and AC3 are displayed in figures S12 A, B, C and S11 G, H, I respectively. See S6 – S8 Figs to visualize the location of the analyzed areas (framed by red squares).

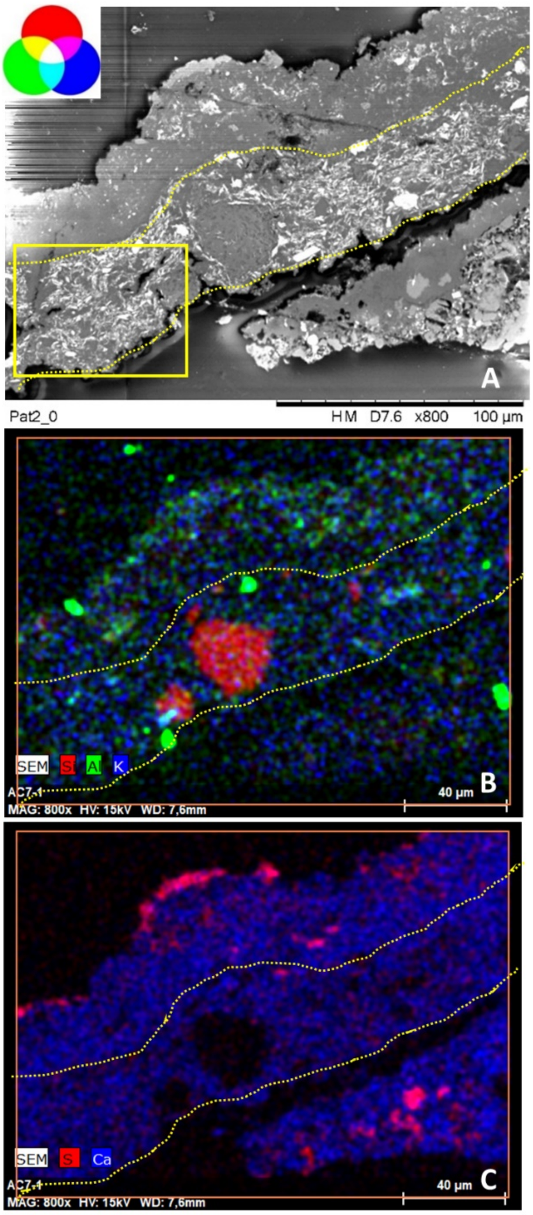

Fig 15. A) SEM backscattered images and their RGB composite images of the elemental distribution of B) $Al_{K\alpha}/Si_{K\alpha}/K_{K\alpha}$ and C) $S_{K\alpha}/Ca_{K\alpha}$ of a selected area of cross-section AC7, constitutive of group 4. See S8 Fig to visualize the location of the analyzed areas (framed by a red square).

Table 2. SEM-EDX quantitative analyses calculated on selected areas (highlighted with yellow circles in the inset SEM figures) of the red pictorial layers of cross-sections AC1 – AC9. The results are reported in percentage of weight normalized to 100 (wt%). The error values have been automatically calculated by the instrumental software (err%). The average EDX spectrum of each area is also reported.

SEM-EDX Quantitative Analyses

AC1bottom

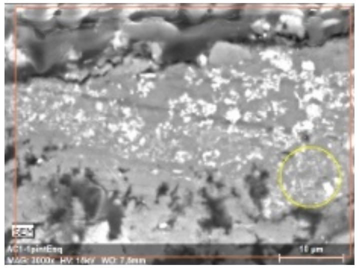

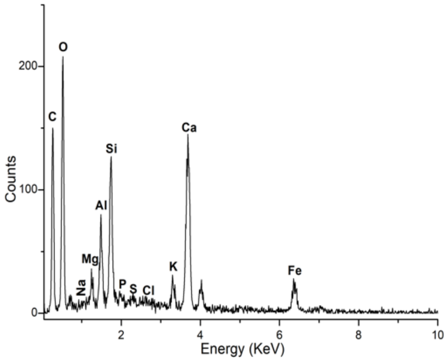

AC1top

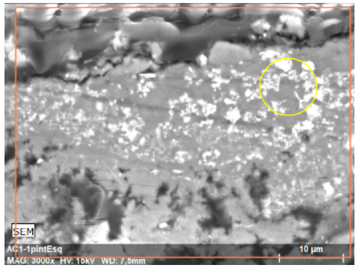

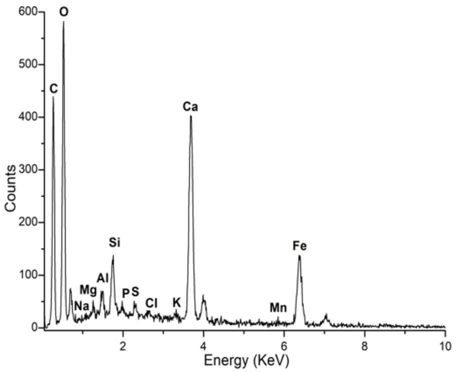

AC2.1

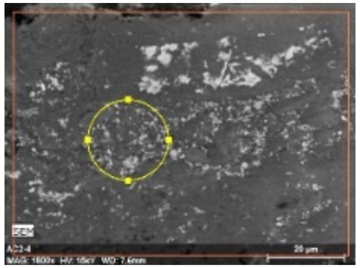

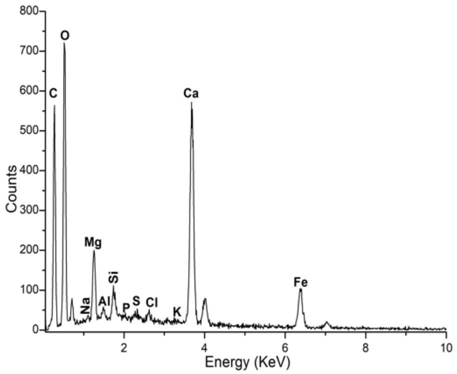

AC2.2

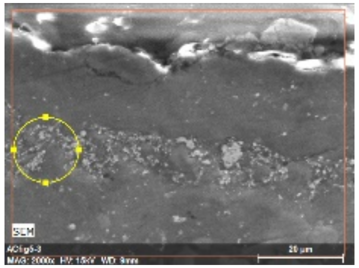

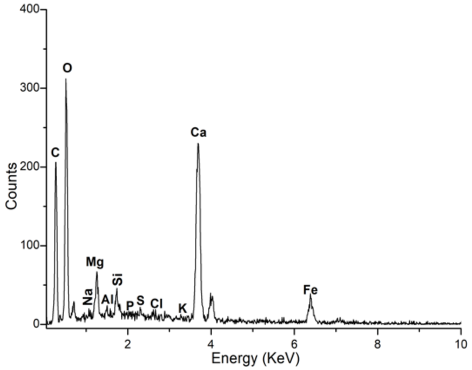

Element	AC1bottom Wt %	AC1bottom Err %	AC1top Wt %	AC1top Err %	AC2.1 Wt %	AC2.1 Err %	AC2.2 Wt %	AC2.2 Err %
**O**	42,73	6,933	41,67	5,739	46,31	6,335	49,07	7,296
**C**	24,16	4,535	23,85	3,598	23,06	3,450	21,71	3,670
**Ca**	13,73	0,4438	14,00	0,4310	15,98	0,5240	17,51	0,5510
**Fe**	**8,413**	**0,2772**	**17,17**	**0,5180**	**9,694**	**0,3226**	**8,234**	**0,2682**
**Mg**	0,9314	0,0755	0,2550	0,03824	2,069	0,1806	2,004	0,1320
**Si**	5,040	0,2398	1,587	0,08982	0,5851	0,06498	0,8052	0,05882
**K**	1,438	0,07010	0,3815	0,03646	0,2527	0,03311	0,06039	0,02686
**Na**	0,1396	0,03387	0,1524	0,001	0,0926	0,03102	0,1606	0,03505
**S**	0,2571	0,03431	0,1822	0,03132	0,2130	0,03290	0,1595	0,03069
**Al**	2,644	0,1506	0,6687	0,05544	0,3116	0,04016	0,1768	0,03328
**Cl**	0,2513	0,03359	0,1714	0,03061	0,2955	0,03533	0,1175	0,02895
**P**	0,2739	0,03567	0,09070	0,02838	0,0610	0,02780	0,06039	0,02687
**Sr**	-	-	-	-	-	-	-	-
**Mn**	-	-	0,07650	0,02717	-	-	-	-

	$AC3_{bottom}$		$AC3_{top}$		$AC4_{bottom}$		$AC4_{top}$	
	**Wt %**	**Err %**	**Wt %**	**Err %**	**Wt %**	**Err %**	**Wt %**	**Err %**

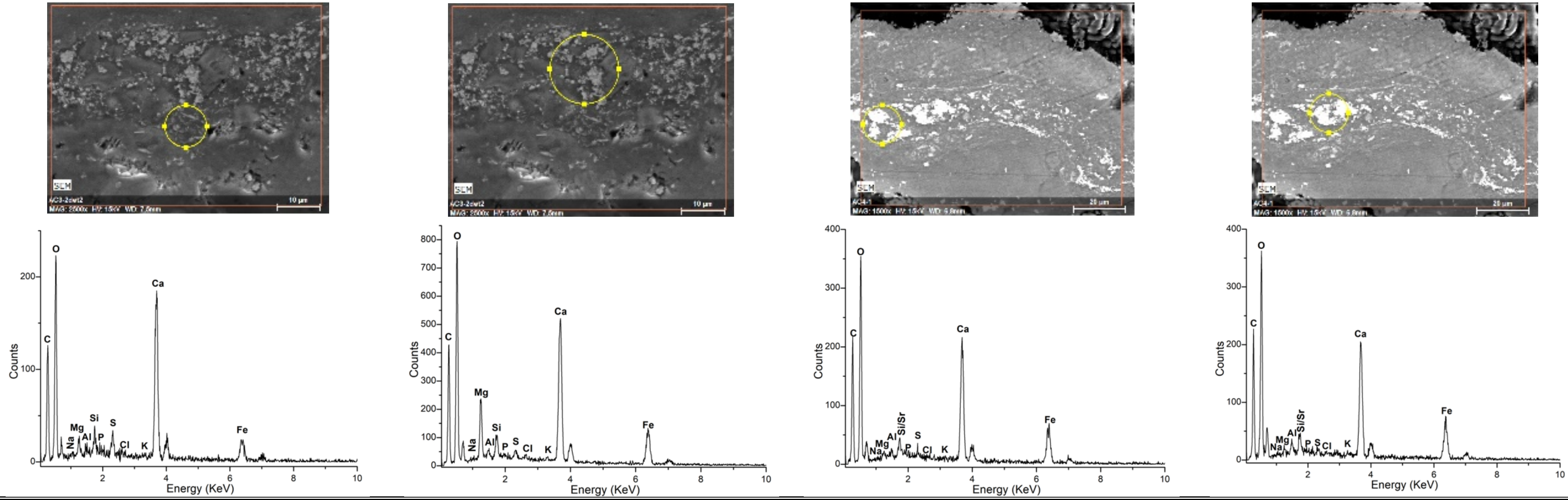

	Wt %	Err %	Wt %	Err %	Wt %	Err %	Wt %	Err %
**O**	48,27	8,043	46,18	6,101	46,41	6,877	46,80	6,935
**C**	19,88	3,948	19,19	2,908	22,20	3,762	21,78	3,720
**Ca**	18,50	0,6136	16,05	0,5116	14,09	0,4550	13,02	0,4233
**Fe**	**8,677**	**0,2965**	**11,73**	**0,3745**	**14,52**	**0,4604**	**15,83**	**0,5012**
**Mg**	0,9740	0,0801	3,810	0,2302	0,2770	0,04008	0,2930	0,4100
**Si**	1,108	0,07428	1,211	0,07633	0,8543	0,06141	0,9650	0,06623
**K**	0,2723	0,03391	0,3217	0,03503	0,2800	0,03376	0,3034	0,03454
**Na**	0,3402	0,04755	0,1834	0,03658	0,06840	0,2934	0,1383	0,03400
**S**	1,152	0,06854	0,4665	0,04180	0,3307	0,03978	0,1760	0,03140
**Al**	0,3785	0,04378	0,4448	0,04601	0,3110	0,03885	0,5243	0,05000
**Cl**	0,2516	0,03396	0,2595	0,03381	0,0980	0,02833	0,03926	0,001303
**P**	0,2020	0,03321	0,1504	0,03082	0,1651	0,03142	0,03657	0,001437
**Sr**	-	-	-	-	0,3333	0,03875	0,0705	0,02800
**Mn**	-	-	-	-	-	-	-	-

AC5.1

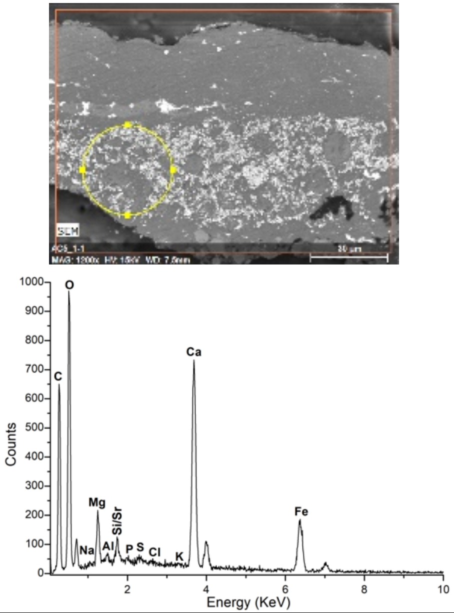

AC5.2

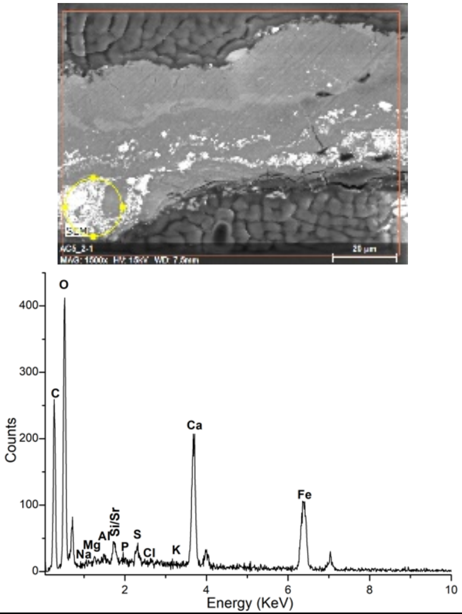

AC6

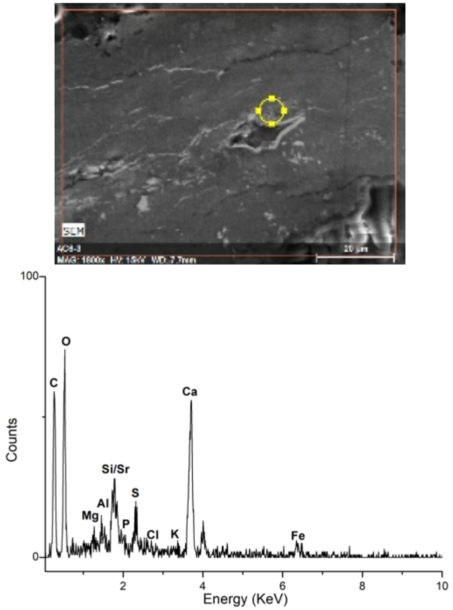

AC7

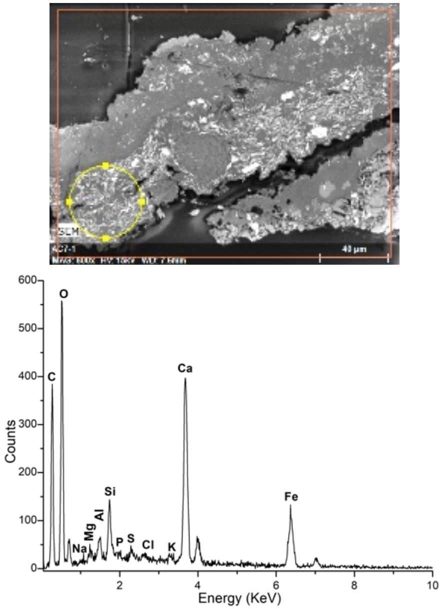

	AC5.1 Wt %	AC5.1 Err %	AC5.2 Wt %	AC5.2 Err %	AC6 Wt %	AC6 Err %	AC7 Wt %	AC7 Err %
**O**	44,57	5,741	43,04	6,369	41,79	8,764	43,60	5,791
**C**	23,39	3,241	21,33	3,654	31,26	6,991	23,00	3,373
**Ca**	14,49	0,4642	11,83	0,3953	14,81	0,4824	14,38	0,4437
**Fe**	**13,20**	**0,4182**	**22,06**	**0,7034**	**3,787**	**0,1399**	**14,38**	**0,4365**
**Mg**	2,207	0,1439	0,1650	0,03418	0,3614	0,04483	0,4265	0,04706
**Si**	0,7657	0,05744	0,6763	0,05458	1,661	0,09666	2,091	0,1101
**K**	0,2180	0,03179	0,1713	0,03051	0,2659	0,03344	0,4375	0,03809
**Na**	0,1382	0,03372	0,04978	0,02824	-	-	0,01611	0,0009769
**S**	0,2555	0,03419	0,5233	0,04444	1,393	0,07605	0,3814	0,03818
**Al**	0,2985	0,03910	0,1399	0,03182	0,5242	0,05022	0,8347	0,06287
**Cl**	0,1682	0,03070	0,006546	0,00022923	0,2756	0,03452	0,2958	0,03464
**P**	0,1349	0,03022	0,0009479	1,788 E-05	0,3602	0,03344	0,1511	0,03062
**Sr**	0,1753	0,03219	-	-	3,519	0,1720	-	-
**Mn**	-	-	-	-	-	-	-	-

AC8bottom	AC8top	AC9
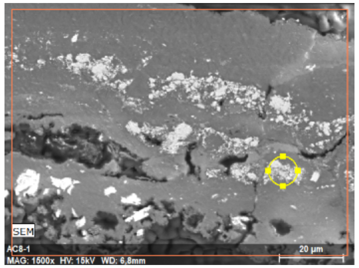	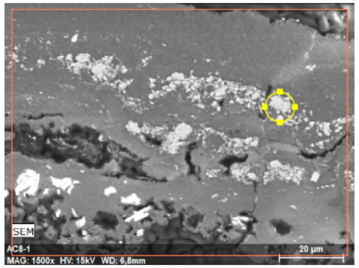	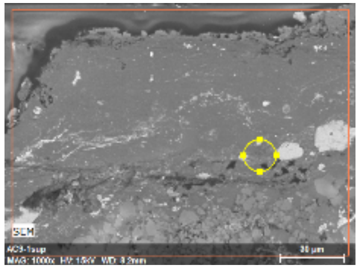
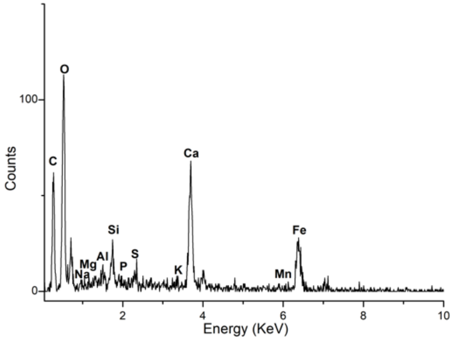	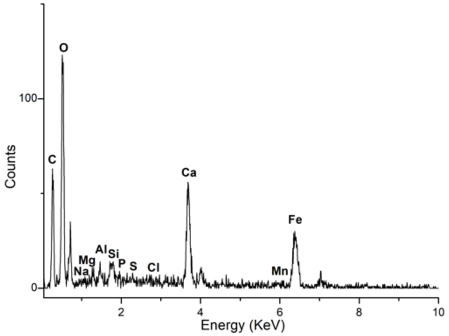	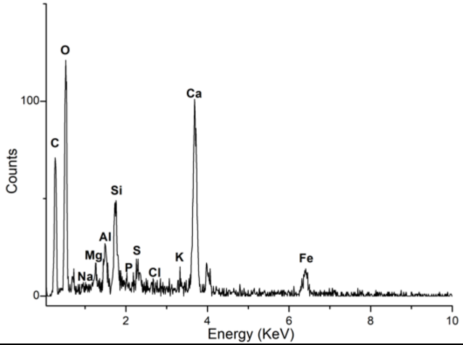

	Wt %	Err %	Wt %	Err %	Wt %	Err %
**O**	45,58	8,310	45,60	8,000	46,69	8,225
**C**	19,64	4,620	20,44	4,545	20,77	4,516
**Ca**	12,35	0,4052	10,55	0,3424	17,87	0,5626
**Fe**	**19,00**	**0,600**	**20,89**	**0,6425**	**7,231**	**0,2388**
**Mg**	0,07000	0,02870	0,3066	0,02818	0,7564	0,06544
**Si**	1,62	0,1000	0,8960	0,06265	3,367	0,1666
**K**	0,2820	0,03400	-	-	0,6128	0,04395
**Na**	0,0464	0,00287	0,1570	0,3483	0,06797	0,02926
**S**	0,5394	0,04471	0,03885	0,00139	0,8209	0,05432
**Al**	0,6047	0,05400	0,2960	0,03893	1,566	0,09842
**Cl**	0,02813	0,0001	0,01820	0,000612	0,09591	0,02823
**P**	0,001	0,0001	0,001	0,0002	0,1448	0,03056
**Sr**	-	-	-	-	-	-
**Mn**	0,3430	0,03525	0,6306	0,04342	-	-

Table 3. Representative Raman signatures (λ_{exc} = 532 and 785 nm) acquired on the red pictorial layers of cross-sections AC1-AC4 collected at el Carche rock shelter. Peaks are observed in cm^{-1}. vs = very strong; s = strong; m = medium; w = weak; vw = very weak; sh = shoulder; br = broad. The values between parentheses are sometimes observed.

Assignation	Samples													
	$AC1_{bottom}$		$AC1_{top}$		AC2.1		AC2.2		$AC3_{bottom}$		$AC3_{top}$		AC4	
λ_{exc}	532 nm	785 nm	532 nm	785 nm	532 nm	785 nm	532 nm	785 nm	532 nm	785 nm	532 nm	785 nm	532 nm	785 nm
Whewellite	-	-	135w	-	135w	-	-	-	-	-	-	-	-	-
Dolomite	-	-	-	-	173m	-	175m	-	(175w)	-	(176m)	-	-	-
Whewellite	-	-	-	-	-	-	-	-	-	-	-	-	-	-
α-quartz	-	-	-	-	-	-	-	-	(204w)	-	-	-	(206w)	-
Hematite	222m	222sh	223w	223s	220m	223s	222s	223s	222m	223m	222s	222s	222m	222s
-	243sh	-	-	243sh	245sh	244sh	244w	244sh	244sh	246sh	242sh	244sh	243sh	242sh
feldspar	(260w)	-	-	-	(264w)	-	-	-	(260w)	-	-	-	-	-
α-quartz	-	-	-	-	-	-	(264w)	-	(264w)	264w	(264s)	-	(264w)	-
Feldspar	(270w)	-	-	-	(270w)	-	-	-	(270w)	-	(270w)	-	(270w)	-
Calcite	-	-	-	(282w)	-	-	-	-	-	-	-	-	-	-
Hematite	290m-br	293m-br	289w	291vs	290m	290vs	290s	290s	290s	290vs	290s	290vs	289m	289vs
Dolomite	-	-	-	-	-	-	300w	-	-	-	-	-	-	-
α-quartz	-	-	-	-	-	-	-	-	(355w)	-	(355w)	-	(351w)	-
-	-	-	-	-	-	-	-	-	-	393w	(393w)	-	(390w)	-
Hematite	406m-sh	407m-br	405w	407s	406m	408s	408s	408s	406m	407s	405s	406s	408m	406s
Feldspar	-	-	-	-	(445w)	-	(453w)	-	-	-	-	-	(453w)	-
α-quartz	-	-	-	-	-	-	(464w)	-	(464w)	464w	-	-	(464w)	-
Feldspar	-	-	-	-	-	-	-	-	(478w)	-	(480w)	-	-	-
Hematite	-	497vw	-	493w	-	496w	495w	494vw	-	497w	499w	494w	-	495w
Feldspar	-	-	-	-	-	-	-	-	(510w)	(514w)	(504w)	-	(508w)	-
-	-	-	-	-	-	-	-	-	-	-	-	-	-	-
Hematite	-	610w	-	608m	-	609m	604w	609vw	607w	609m	608w	609m	-	608m
-	-	647vw	-	650sh-vw	-	653vw	-	650sh-vw	-	650w	-	645sh-vw	-	650sh-vw
Calcite	-	(712vw)	-	-	-	-	-	-	-	(715vw)	-	-	-	-
Dolomite	-	-	-	-	723w	722w	-	-	-	-	-	-	-	-
Whewellite	895w	894w	-	894w	893w	(893w)	-	894vw	(895w)	895w	(895w)	895vw	-	893w
Apatite	-	-	-	-	-	-	-	-	-	-	-	-	960m	-
Celestite	-	-	-	-	-	-	-	-	-	-	-	(1001w)	-	(1000vw)
Gypsum	-	-	1006w	-	-	-	-	-	-	(1008w)	-	-	-	-
Calcite	-	(1089vw)	-	(1086w)	-	-	-	-	-	(1089vw)	-	-	-	(1086vw)
Dolomite	-	-	-	-	1096m	1096m	1095s	1095w	-	-	(1096m)	1095w	-	-
Feldspar	-	-	-	-	-	-	-	-	-	-	-	-	(1126w)	-
α-quartz	-	-	-	-	-	-	-	-	(1162w)	-	-	-	-	-
Am. Carbon	-	-	-	-	-	-	-	-	(1360s)	(1317s)	-	-	-	-
Hematite	(1318w)	-	-	-	1320w	-	1320vs	-	1320s	-	1316s	-	1315m	-
-	-	-	-	-	-	-	-	-	-	-	-	-	-	-
Whewellite	1461w	1460w	1460w	-	1464w	1461w	1463m	1460w	1460w	1459w	(1462w)	1461w	-	1460w
Weddellite	(1473w)	-	-	-	-	-	-	-	-	-	(1472w)	(1470w)	-	-

**Whewellite**	1486w	1483w	1488w	-	1486w	1485w	1486m	1488w	1486w	1484w	(1484w)	1488w	-	1482w
**Am. Carbon**	-	-	-	-	-	-	-	-	(1601s)	(1577s)	-	-	-	-
**Whewellite**	1628w	-	-	-	-	-	-	-	-	-	-	-	-	-

Table 4. Representative Raman signatures (λ_{exc} = 532 and 785 nm) acquired on the red pictorial layers of cross-sections AC5-AC9 collected at el Carche rock shelter. Peaks are observed in cm^{-1}. vs = very strong; s = strong; m = medium; w = weak; vw = very weak; sh = shoulder; br = broad. The values between parentheses are sometimes observed. In sample AC9, a spectrum acquired in a black crystal present in the pictorial layer showed the same peaks but much more intense. In sample AC6 the fluorescence signal covered any possibility to analyze the cross-section using 532 nm excitation laser.

**Assignation**	**Samples**											
	**AC5.1**		**AC5.2**		**AC6**		**AC7**		**AC8**		**AC9**	
λ_{exc}	**532 nm**	**785 nm**	**532 nm**	**785 nm**	**532 nm**	**785 nm**	**532 nm**	**785 nm**	**532 nm**	**785 nm**	**532 nm**	**785 nm**
**Whewellite**	-	-	-	-	-	-	135w	-	-	-	-	-
**Dolomite**	-	-	-	-	-	-	-	-	-	-	-	-
**Whewellite**	-	-	-	-	-	-	190w	-	-	-	-	-
**α-quartz**	-	-	-	-	-	-	(202w)	-	-	-	-	-
**Hematite**	**223m**	**223s**	**223s**	**223s**	-	**224w**	**222s**	**225s**	**222s**	**223m**	**222m**	**223w**
**-**	**243sh**	**243w**	**243sh**	**242sh**	-	-	**243s**	-	**245m**	**246sh**	**242sh**	**245sh**
**feldspar**	(264w)	-	-	-	-	-	(206w)	-	(260w)	-	(258w)	-
**α-quartz**	-	-	(264w)	-	-	-	(264w)	-	-	264w	(264w)	-
**Feldspar**	(285w)	-	-	-	-	-	(270w)	-	-	-	(270w)	-
**Calcite**	-	-	-	-	-	-	-	-	-	--	-	-
**Hematite**	**290s**	**291vs**	**290s**	**290vs**	-	**292m-br**	**290s**	**293vs**	**288s**	**290vs**	**289m**	**291w**
**Dolomite**	-	-	-	-	-	-	-	-	-	-	-	-
**α-quartz**	(351w)	-	(355w)	-	-	-	-	-	(355w)	-	(355w)	-
**-**	-	-	-	-	-	-	-	-	-	393w	-	-
**Hematite**	**409m**	**407s**	**406s**	**407s**	-	**407m-br**	**408vs**	**411vs**	**407s**	**407s**	**406s**	**410w**
**Feldspar**	(453)	-	-	-	-	-	(445w)	-	(453w)	-	-	-
**α-quartz**	-	-	(464w)	-	-	-	(464w)	-	(464w)	464w	(465w)	-
**Feldspar**	(473)	-	-	-	-	-	-	-	(482w)	-	(473w)	-
**Hematite**	493w	**495w**	**500w**	**495w**	-	**495vw**	**498m**	**495m**	**496w**	**497w**	**495m**	**495vw**
**Feldspar**	(512)	-	-	-	-	-	(515w)	-	(510w)	(514w)	(512w)	-
**-**	-	-	-	-	-	-	(584w)	-	-	-	-	-
**Hematite**	**609s**	**607w**	**608w**	**608m**	-	**609m**	**607s**	**610s**	**607w**	**609m**	**606m**	**609w**
**-**	-	**652vw**	-	**650w-sh**	-	-	-	**650sh-vw**	-	**650sh-vw**	-	**650sh-vw**
**Calcite**	-	-	-	-	-	-	-	(716w)	-	-	-	-
**Dolomite**	-	-	-	-	-	-	-	-	-	-	-	-
**Whewellite**	-	-	(891w)	894w	-	895w	894w	894vw	894m	895w	894w	-
**Apatite**	960vw	-	(960w)	-	-	-	-	(960w)	-	961w	-	-
**Celestite**	-	(1001w)	-	-	-	(999w)	-	-	(997w)	-	-	-

**Gypsum**	-	-	-	-	-	-	-	-	-	(1008w)	-	-
**Calcite**	-	-	-	(1085vw)	-	(1088w)	-	(1088w)	-	-	-	-
**Dolomite**	(1093vw)	-	-	-	-	-	-	-	-	-	-	-
**Feldspar**	(1123)	-	-	-	-	-	-	-	-	-	(1122w)	-
**α-quartz**	-	-	-	-	-	-	-	-	-	-	-	-
**Am. Carbon**	-	-	-	-	-	(1318w)	-	-	(1349m)	(1317s)	-	-
**Hematite**	**1317m**	-	**1320s**	**-**	-	-	**1315vs**	**-**	**1320vs**	**-**	**1315s**	**-**
**Organic***	-	-	-	(1445w)	-	-	-	-	-	(1445w)	-	-
**Whewellite**	(1462w)	-	-	1458w	-	1460m	1462w	1460m	1461m	1459w	-	1460w
**Weddellite**	-	-	-	(1473w)	-	(1476vw)	-	-	-	-	-	-
**Whewellite**	(1488w)	-	(1486vw)	1482w	-	1487m	1485w	1486m	1486m	1484w	1488w	1488w
**Am. Carbon**	-	-	-	-	-	(1585w)	-	-	(1601m)	(1577s)	-	-
**Whewellite**	-	-	-	1626w	-	-	-	-	1626w)	-	-	-
**Organic**	(1704w)	-	-	-	-	-	-	-	1724w	-	-	-

*the signal can be related to a calcium carboxylate specie [6].

References

[1] Miliani C, Rosi F, Daveri A, Brunetti BG. Reflection infrared spectroscopy for the non-invasive in situ study of artists' pigments. Appl Phys A. 2012;106: 295–307. DOI 10.1007/s00339-011-6708-2.

[2] Bishop JL, King SJ, Lane MD, Brown AJ, Lafuente B, Hiroi T, et al. Spectral properties of anhydrous carbonates and nitrates. Earth and Space Science. 2021; 8. e2021EA001844. Doi:10.1029/2021EA001844.

[3] Monico L, Rosi F, Miliani C, Daveri A, Brunetti BG. Non-invasive identification of metal-oxalate complexes on polychrome artwork surfaces by reflection mid-infrared spectroscopy. Spectrochim Acta A Mol Biomol Spectrosc. 2013;116: 270–280.

[4] Bishop J, Lane MD, Darby Dyar M, King SJ, Brown AJ, Swayze GA. Spectral properties of Ca-sulfates: Gypsum, bassanite, and anhydrite. American Mineralogist. 2014;99: 2105–2115.

[5] Kloprogge JT, Ruan H, Duong LV, Frost RL. FT-IR and Raman microscopic study at 293 K and 77 K of celestine, $SrSO_4$, from the middle triassic limestone (Muschelkalk) in Winterswijk, The Netherlands. Neth J Geosci. 2001;80(2): 41-47.

[6] Otero V, Sanches D, Montagner C, Vilarigues M, Carlyle L, Lopes JA et al. Characterisation of metal carboxylates by Raman and infrared spectroscopy in works of art. J Raman Spectrosc. 2014; 45: 1197–1206.
